# Conserved innate immunity components limit transgene expression in adult planarians

**DOI:** 10.1242/dev.205222

**Published:** 2026-03-27

**Authors:** Leonard Drees, Daniel Blösel, Johannes Kowalewski, Uri Weill, Jochen C. Rink

**Affiliations:** ^1^ Department of Tissue Dynamics and Regeneration, Max Planck Institute for Multidisciplinary Sciences, 37077 Göttingen, Germany; ^2^ Faculty of Computing, Mathematics, Engineering and Natural Sciences, Northeastern University London, London E1W 1LP, UK; ^3^ Faculty of Biology und Psychology, Georg-August-University Göttingen, 37073 Göttingen, Germany

**Keywords:** Immune response, Planaria, Transfection, Reporter expression

## Abstract

The planarian flatworm *Schmidtea mediterranea* has become a powerful model for studying whole-body regeneration, tissue patterning, and stem cell regulation. Yet the absence of reliable tools for transgene expression still limits the elucidation of molecular mechanisms in in this system. Here, we establish a proof-of-principle system for plasmid-based expression of NanoLuciferase (NanoLuc) in *S. mediterranea*, employing commercially available transfection reagents and a panel of endogenous promoter sequences. Despite successful delivery, reporter expression remained low and transient. To identify biological barriers to robust transgene expression, we investigated the role of innate immune pathways. Candidate gene searches and biochemical pull-down of cytoplasmic DNA coupled to mass spectrometry identified several planarian homologs of conserved immune regulators and putative DNA sensors. Through RNA interference screening of conserved innate immune components, we uncover roles for *S. mediterranea* homologs of Tank-binding kinase 1 (TBK1) and macrophage mannose receptor 1 (MRC1) as potent repressors of transgene expression. Transcriptomic and functional analyses further implicate TBK1 in regulating broad innate immune and stress-response programs, akin to its vertebrate function. Together, our findings demonstrate that innate immune signaling limits transgene expression in *S. mediterranea* and suggest that modulating these pathways may be key to enabling stable and efficient genetic manipulation in planarians.

## Teaser

**Highlighted Article:** Plasmid-based NanoLuc expression in planaria is used to identify innate immune components that limit transgene expression, providing proof of principle for a transgenic approach that could be useful in this model organism.

## INTRODUCTION

Planarian flatworms are fascinating animals. Many planarian species have the ability to regenerate their whole body even from small pieces of tissue (Morgan, 1898; Newmark and Sánchez Alvarado, 2001). Other planarian species have various regeneration defects or cannot regenerate at all (Vila-Farré et al., 2023; Vila-Farré and Rink, 2018). In addition, planarians do not reach a stable body size, but grow when fed and shrink when starved, succeeding in scaling all organs and constituting cells over a tremendous range of body sizes (Ivankovic et al., 2019; Thommen et al., 2019; Baguñà, 1976; Guo, 2025). Other fascinating phenotypic traits of planarians include a wide range of reproductive strategies and probably life expectancies (Vila-Farré et al., 2023; Drees and Rink, 2023). On a cell biological level, planarian regeneration depends on abundant adult pluripotent stem cells – so called neoblasts – which are the only division-competent cells outside of the reproductive system (Newmark and Sánchez Alvarado, 2000; Baguñà, 2012; Kashima et al., 2020; Reddien, 2018). The two planarian species *Schmidtea mediterranea* (*Smed*) and *Dugesia japonica* have been developed into molecularly tractable laboratory models. The current tool kit for studying this unique biology includes an annotated chromosome-scale reference genome assembly (Ivanković et al., 2024), single-cell sequencing gene expression atlases (Fincher et al., 2018; Plass et al., 2018), robust whole-mount *in situ* RNA hybridization and immunostaining methods for visualizing gene products (Grohme et al., 2023; King and Newmark, 2013; Ross et al., 2015) as well as fluorescent-activated cell sorting (FACS) of specific cell populations (Romero et al., 2012; Higuchi et al., 2007). A systemic RNA interference (RNAi) response upon feeding of dsRNA and the use of small molecule inhibitors are available for the analysis of gene functions (Newmark et al., 2003; Rouhana et al., 2013; Oviedo et al., 2010; Zhang et al., 2011). What remains lacking, despite substantial efforts over the years, are effective tools for transgene expression.

Our recent demonstration of mRNA-based transfection and expression of a Nano-Luciferase (NanoLuc) transgenic reporter in *Smed* (Hall et al., 2022) provided an important proof of principle for transgene expression in this planarian model species. While the protocol allows the robust detection of reporter expression due to the very low background of the luciferase reaction (England et al., 2016), the currently achievable expression levels remain insufficient for other reporter systems, e.g. commonly used fluorescent proteins or immunostaining methods.

Generally, transgene expression necessitates the efficient transfer of the transgene encoding vector across the plasma membrane and into the cytosol for ribosomal translation (mRNA transfection) or additionally into the nucleus for transcription in the case of DNA-based vectors. Low or no transgene expression can consequently be the result of multiple technical issues. In addition, biological barriers can limit transgene expression. In many animals, the transfection of nucleic acids inadvertently triggers anti-viral responses of the innate immune system (Warga et al., 2023; Chattergoon et al., 1998). Viral nucleic acids can be sensed already at the plasma membrane by highly conserved Toll-like receptors (TLRs) and RIG-I-like receptors (RLRs) (Leulier and Lemaitre, 2008; Zou et al., 2009). Specific TLRs and RLRs sense ssRNA, rRNA, dsRNA or CpG dsDNA and elicit appropriate activation of innate immune pathways (Takeda and Akira, 2015; Kumagai et al., 2008). In the cytoplasm, cyclic GMP-AMP synthase (cGAS) and AIM2 are conserved sensors of dsDNA (Fernandes-Alnemri et al., 2009; Civril et al., 2013). Once activated by binding to their respective target, these sensors elicit downstream signaling cascades via kinases or, in the case of cGAS, via production of the second messenger cyclic GMP-AMP (cGAMP) that activates STING (stimulator of interferon genes) (Ablasser et al., 2013). Multiple anti-viral and anti-microbial sensing pathways converge on the activation of Tank binding kinase 1 (TBK1), which is a central hub for activation of transcriptional regulators such as nuclear factor kappa B (NF-κB) and interferon regulator transcription factors (IRFs) 3 or 7 (Zhou et al., 2020). The transcriptional response includes cell-autonomously acting stress regulators that can broadly suppress translation within the cell or trigger cell death pathways, but also pro-inflammatory cytokines including interleukins and anti-viral defense proteins that act non-cell-autonomously and can trigger inflammation and organism-wide responses to limit infection (Paludan and Bowie, 2013). Transgene expression protocols in model organisms therefore often target embryonic stages at which these pathways are often not active yet (Tran and Luallen, 2024; Guo, 2019).

Currently, comparatively little is known about the innate immune system of planarians. A planarian homolog of the dsRNA-sensing RIG-I receptor has been characterized as an innate immune response regulator (Li et al., 2019), a specialized cell type called ‘ruptoblasts’ was shown to be involved in immune defense (Chai et al., 2025 preprint), and the stress response to bacterial infection has been shown to depend on MAP kinase and p38 signaling (Arnold et al., 2016) and phagocytosis involving Morn2 (Abnave et al., 2014). This suggests that nucleic acid sensing and general innate immunity pathways are conserved and active in adult planarians. At the same time, the investigation of planarian embryogenesis is hindered by the small size of the zygotes, their embedding within compact yolk cell masses inside a rigid egg shell (Stevens, 1904; Steiner et al., 2016; Best et al., 1969; Davies et al., 2017; Alvarado, 2003; Cardona et al., 2006), and by the widespread use of clonal strains that do not produce gametes (Issigonis and Newmark, 2019; Drees and Rink, 2023). Together, these factors suggest that active innate immune barriers may significantly contribute to the persistent challenge of establishing stable transgene expression in this system.

Here, we advance efforts to establish transgenic methods in planarians by providing proof of principle for plasmid-based NanoLuc reporter expression. We identified several endogenous regulatory regions, including both housekeeping and stem cell-specific promoters, that successfully drive robust transgene expression in *Smed*. However, the expression levels invariably remained similarly low as those achieved using previously described mRNA transfection methods (Hall et al., 2022). We therefore investigated intrinsic barriers to efficient transgene expression. Motivated by our observation that DNA transfection can trigger planarian fission, we screened a small set of conserved innate immunity genes and biochemically identified additional candidate DNA-binding proteins in transfected animals. Notably, RNAi-mediated knockdown of *TBK1* and *Macrophage Mannose Receptor 1* (*MRC1*) significantly increased NanoLuc reporter expression. Furthermore, we show that TBK1 is a key regulator of both cell-autonomous and non-autonomous innate immune signaling in planarians and therefore a deeply conserved node in innate immune signaling. Our demonstration that the suppression of innate immunity pathways enhances transgene expression identifies a biological barrier to genetic manipulation in the system and a new research avenue towards the goal of stable transgenesis in planarians.

## RESULTS

### *In vivo* transfection with NanoLuc reporter plasmids

**Fig. 1.**
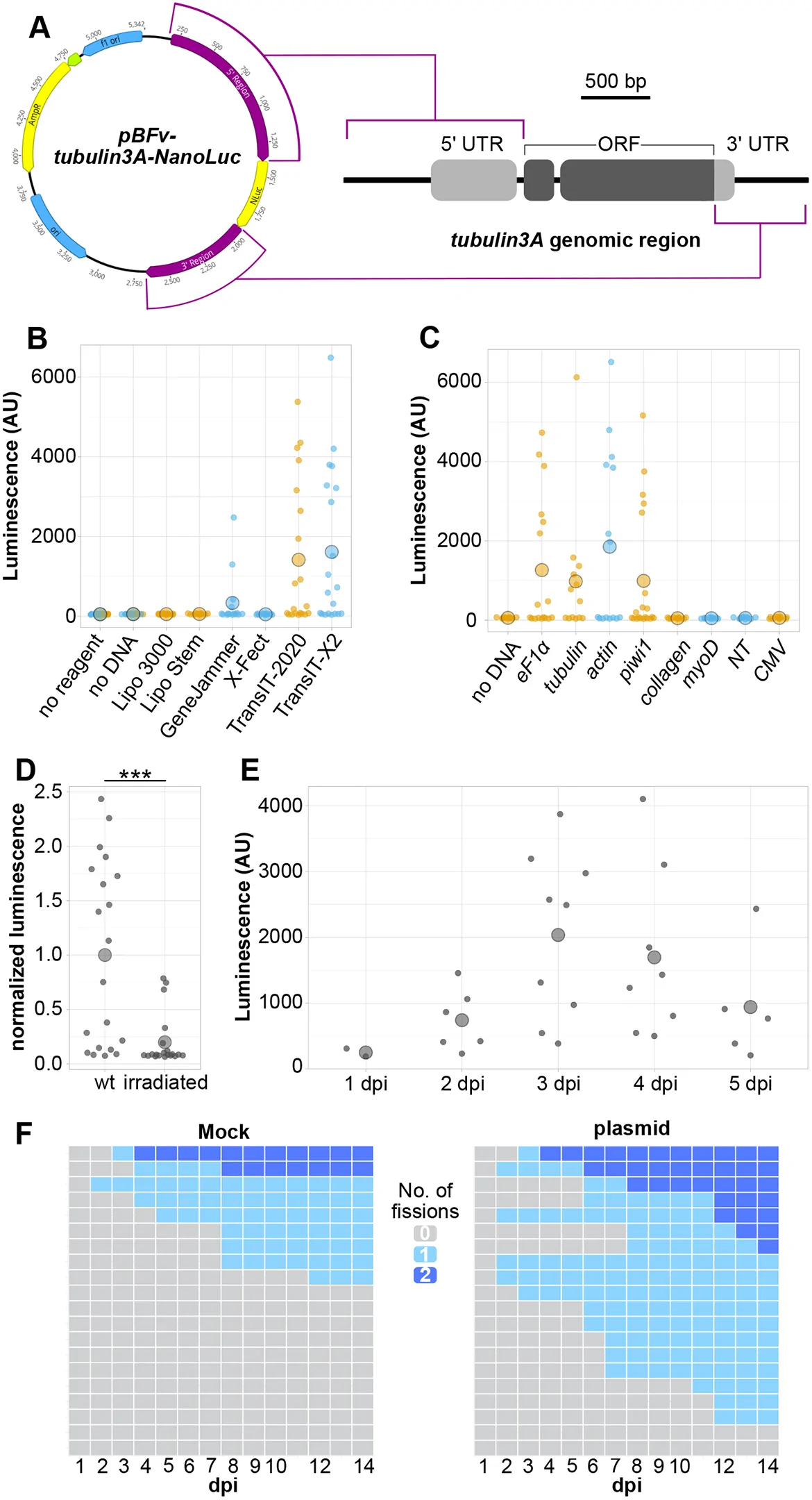
**Plasmid-based transgene expression in *Smed.*** (A) Schematic of a NanoLuc reporter plasmid harboring 5′ and 3′ genomic regions of the *tubulin3A* gene. Endogenous UTRs (light gray) flank the NanoLuc ORF that replaces the endogenous ORF encoding exons (dark gray). (B,C) Luminescence signals of worms injected with *pBFv-eF1a-NanoLuc* plasmid and the indicated transfection reagents (B) or reporter plasmids with the indicated regulatory regions and TransIT-X2 (C) or no DNA at 3 days after injection/transfection. Each small data point represents signal of a single worm and large data points the mean value of the group. Colors indicate groups that were processed in parallel in one experiment. Control groups from different batches for ‘no reagent’ and ‘no DNA’ are shown in the same column. (D) Luminescence signals of wild-type (wt) and lethally irradiated worms (60 Gray) at 5 days post-irradiation normalized to the mean of wt. Worms were injected with *pBFv-eF1a-NanoLuc* plasmid and TransIT-X2 reagent. Measurements were taken 3 days after injection/transfection. ***P=0.00012 (unpaired *t*-test). (E) Luminescence signals of individual worms injected with *pBFv-eF1a-NanoLuc* plasmid and TransIT-X2 reagent at different days post-injection (dpi). (F) Quantification of fission events after injection of mock (TransIT-X2, no DNA; left) or complete transfection mix (TransIT-X2 plus reporter plasmid; right). Individual worms (rows) were followed for 14 dpi (columns). Light blue shading, first fission; dark blue shading, subsequent fission. AU, arbitrary units.

While mRNA-based reporters simplify transgene gene expression by bypassing the complexities of transcription, they are limited by the short half-life of mRNAs and lack the potential signal amplification that transcription contributes. Motivated by these considerations, we designed DNA-based NanoLuc reporter constructs on basis of our previously established mRNA-based NanoLuc reporter and transfection protocols. For the selection of putative promoter sequences, we queried PlanMine (Rozanski et al., 2019) for genes with particularly high expression levels either in a broad range of cell types (‘housekeeping genes’; *eF1α*, *tubulin*, *actin*), in differentiated cell types (muscles and epidermis; *collagen* and *neurotrypsin*) or in the pluripotent adult stem cells of planarians (neoblasts) as particularly strategic targets for transgenesis approaches (*piwi-1*; Table S2). Taking advantage of the recent revision of the *Smed* genome assembly, genome annotations and multi-species ATACseq data tracks (Ivanković et al., 2024), we selected and cloned 800-2000 bp regions upstream and downstream of the respective genes’ open reading frames (ORFs) as likely regulatory sequences. Only the ORF-encoding exons were replaced by a *Smed* codon-optimized version of the NanoLuc ORF (Fig. 1A; Table S3) and assembled into a series of expression vectors. Starting with the *eF1α*-NanoLuc reporter plasmid, we screened a range of commercially available transfection reagents and injected the respective transfection mixtures between the two posterior gut branches as customary live worm injection site as described for cell transplantation and mRNA transfection methods (Hall et al., 2022; Wang et al., 2018). Initial transfections of digoxigenin (DIG)-labeled plasmid with and without transfection reagent revealed that transfection reagent restricts dispersion and keeps the plasmid concentrated at the injection site (Fig. S1A). However, the rapid loss of the DIG-DNA signal within 24 h, irrespective of the presence of transfection reagent, suggests the rapid degradation of the bulk of injected plasmid (Fig. S1B).

To assay for the expression of NanoLuc in plasmid-injected worms, lysates of individually dissociated worms were measured 3 days post-injection using a standardized NanoLuc luminescence detection kit on a plate reader. Strikingly, transfection with TransIT-2020 and TransIT-X2 reagents reliably generated luminescent signals in about 50% of the injected worms that were orders of magnitude above that of non-transfected control worms or worms that were only injected with DNA (Fig. 1B). With the few weakly positive animals in GeneJammer transfections as the only exception, all other transfection reagents remained negative (Fig. 1B). These results established a proof of principle of plasmid-based reporter expression in planarians and provided a positive control for further optimizations.

Next, we explored the cell type and promoter/regulatory region dependency of the NanoLuc signal, using the TransIT-X2 reagent for transfection and our previously generated plasmid series with different putative promoter sequences. The injection of plasmids with regulatory regions from the ubiquitously and highly expressed genes *eF1α*, *tubulin* and *actin* all generated NanoLuc signals of similar magnitude and frequency (Fig. 1C). Also, regulatory regions of *piwi-1*, a neoblast marker that is specifically expressed in the stem cell compartment (Reddien et al., 2005), generated NanoLuc expression (Fig. 1C). However, regulatory regions from genes that are specifically expressed in differentiated (non-dividing) cell types, including *neurotrypsin* (*NT*; epidermis), *myoD* (longitudinal muscles) and *collagen* (all muscles), or the *CMV* promoter that is used for high expression levels in mammalian cells (Boshart et al., 1985), did not generate detectable NanoLuc expression (Fig. 1C). These results demonstrate specificity in the promoter activity of the tested sequences and their cell-type specificity further suggests transfection and/or transgene expression enrichment in neoblasts. Indeed, we found that the NanoLuc reporter intensity and transfection efficiency were both strongly reduced when worms were transfected with the *eF1α-NanoLuc* plasmid after neoblast ablation by irradiation (Fig. 1D), thus providing additional evidence that our transfection protocol targets this important cell type. We next assayed the duration of reporter expression by a time-course experiment. The *eF1α*-*NanoLuc* signal peaked at day 3 and declined to baseline levels by day 5 of chase (Fig. 1E). The observed kinetics were very similar to our previous results with mRNA reporters, indicating that the plasmid-based reporters did not substantially increase the time duration of reporter expression (Hall et al., 2022). Similarly, we found that also the reporter expression levels achievable with the *eF1α* promoter and other promoters remained insufficient for the detection of fluorescent reporter proteins (e.g. mCherry or mScarlet).

However, during the course of many NanoLuc expression experiments, we observed that cohorts of worms that had undergone DNA transfection consistently displayed a higher frequency of fission events compared to control groups (Fig. 1F). Notably, this effect was not observed when a mock transfection mix without DNA was injected, ruling out the injection wound or adverse responses to the transfection reagent as triggers of fission initiation. The fact that fission was elicited exclusively by the combination of DNA and transfection reagent demonstrates that planarians sense and respond to transfected DNA, possibly including the suppression of transgene expression.

### Identification of putative innate immunity components

**Fig. 2.**
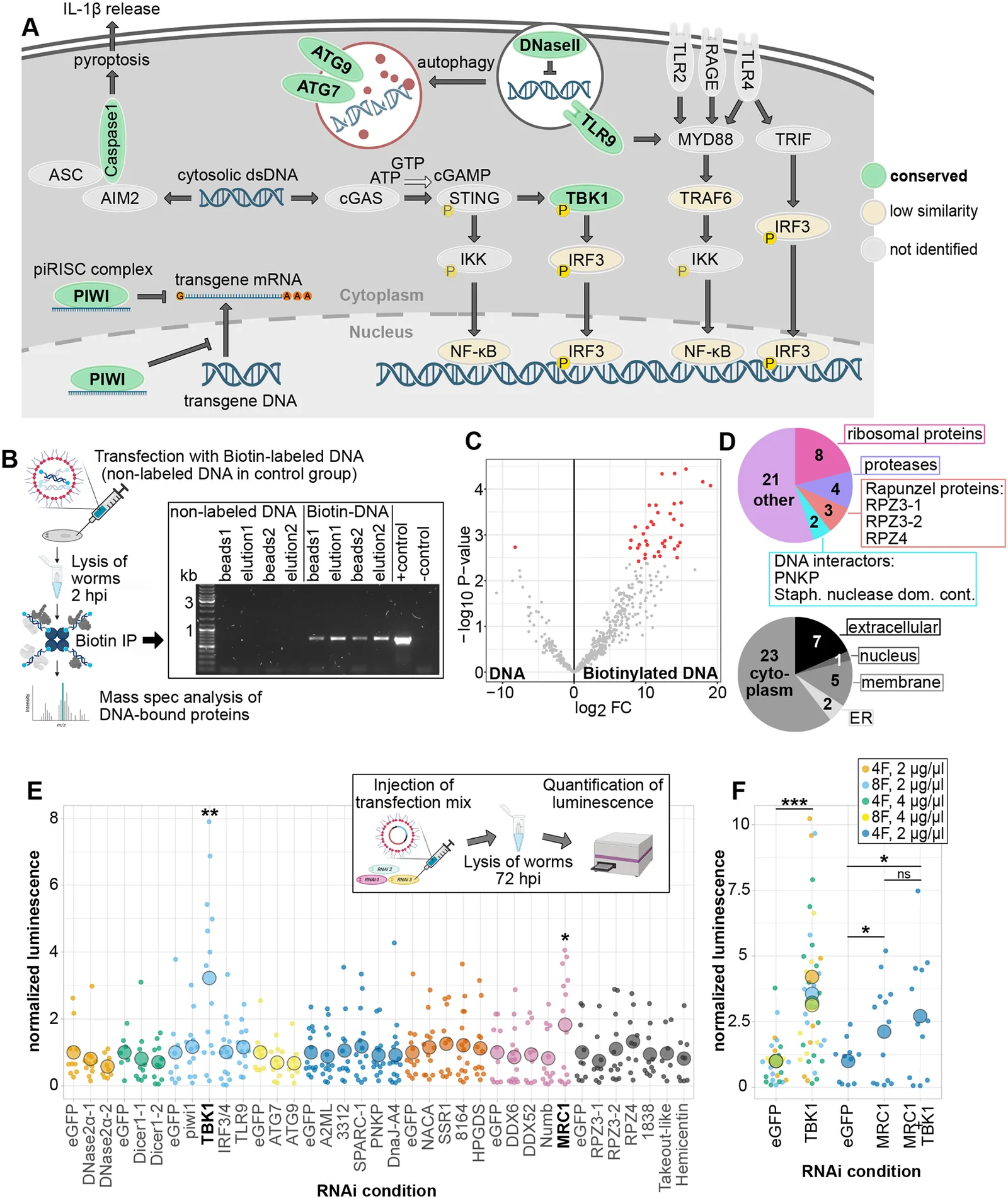
**Identification of TBK1 and MRC1 as suppressors of transgene expression.** (A) Schematic of nucleic acid-sensing and suppression pathways and their conservation in *Smed*. Clearly conserved components are shown in green, putative homologs in yellow and likely absent components in gray. Bold text indicates genes that were functionally analyzed by RNAi (see below). (B) Experimental schematic of biochemical pull-downs of biotin-labeled transfected DNA. The specificity of the pull-down was validated by PCR on the indicated samples (right). (C) Mass spectrometry analysis of pull-downs of biotin-labeled or unlabeled DNA-injected worms. Approximately 40 proteins (red data points in the volcano plot) were significantly enriched in the biotin-pull downs (adjusted *P*<0.05). (D) Analysis of enriched proteins, on the basis of sequence features (top) and predicted subcellular localization [DeepLOC 2.0 predictions (Thumuluri et al., 2022); bottom]. Staph. nuclease dom. cont., staphylococcal nuclease domain-containing protein. (E,F) Functional screen for transgene expression suppression. Worm cohorts fed with dsRNA against the indicated genes (three feedings) were injected with NanoLuc reporter plasmid 3 days before luminescence signal quantification. Values for single worms (small data points) and group mean (large data points) are shown. Measurements were normalized to the mean luminescence signal of the corresponding control (*eGFP*) RNAi group. Bold text in E indicates statistically significant luminescence increase (TBK1 *P*=0.00582; MRC1 *P*=0.02594; one-way ANOVA). In E, colors indicate experimental groups that were processed in independent experiments. In F, colors mark differences in the dsRNAi feeding regiments as indicated and the *P*-values of the marked comparisons are: TBK1-eGFP ***P=0.00006; MRC1-eGFP *P=0.03855; MRC1+TBK1-eGFP *P=0.015649; MRC1+TBK1-MRC1 *P*=0.24405 (ns, not significant) calculated by one-way ANOVA.

In many animals, the innate immune system carries out the sensing of ectopic DNA. Multiple innate immunity pathways are known that sense and respond to DNA in the cytoplasm as a pathogen-associated pattern. However, planarian innate immunity pathways have not been systematically analyzed so far. Starting with an *in silico* approach, we therefore systematically searched the *Smed* transcriptomes and genome annotations for conserved homologs of nucleic acid-sensing pathways and their downstream effectors (see Materials and Methods). In addition to the known conservation of PIWI and Dicer as potential degraders of foreign nucleic acids (Aliyari and Ding, 2009), our search identified homologs of the endosomal DNA receptor TLR9 (Kumagai et al., 2008), the kinase TBK1, which is known to integrate several innate immunity pathways (Zhou et al., 2020), and a diverged homolog of the interferon regulatory transcription factor IRF3 (Table S1; Al Hamrashdi and Brady, 2022). In vertebrates, IRF3 and NF-κB are key mediators of the transcriptional response downstream of TBK1 and the role of NF-κB transcriptions factors is also conserved in arthropods (Al Hamrashdi and Brady, 2022; Msweli et al., 2024). Generally, NF-κBs are member of the Rel homology domain (RHD) transcription factor family, which includes key regulators of immune gene expression in invertebrates and vertebrates (Msweli et al., 2024). While RHD transcription factors are present in Lophotrochozoa, the phylum to which planaria belong, direct NF-κB homologs have proven difficult to identify and only tentative functional links between RHD-containing proteins and innate immunity have been reported (Msweli et al., 2024; Zhang and Coultas, 2011). Concordently, our sequence homology search identified divergent RHD-containing genes in *Smed* with low similarities to immune regulatory NF-κB proteins, while the RHD containing homolog of NFAT5, which does not have a role in immune gene regulation, shows high conservation (Fig. S4C). Furthermore, we also failed to identify homologs of multiple deeply conserved nucleic acid-sensing pathways. Surprisingly, this list included cGAS, a cytoplasmic DNA immune sensor of DNA that is broadly conserved across animals and originates from prokaryotic immune defense systems (Slavik and Kranzusch, 2023), but also the Toll-like Receptors (TLRs) 2 and 4 and RAGE (Fig. 2A). The concomitant absence of homologs of the interaction partners of these DNA sensors, including STING, ASC, MYD88 and TRIF, in our survey (Fig. 2A) suggests that multiple nucleic acid-sensing pathways of the innate immune system appear to be lost in *Smed*, providing a further example of the unusual gene complement of these early branching organisms (Grohme et al., 2018). Reasoning that planarians might have adapted or evolved other DNA sensors, we complemented the *in silico* approach with an experimental screen for DNA binders via transfection of biotin-labeled DNA and subsequent proteomic analysis of proteins pulled down together with the DNA (Fig. 2B). Analysis of the proteins that were uniquely enriched in the immunoprecipitation (IP) of biotin-labeled DNA compared to control pull-downs of non-biotinylated DNA (Fig. 2C; Tables S1 and S6) included the homologs of two bona fide DNA-interacting proteins (PNKP and a staphylococcal nuclease domain containing protein), three homologs of Rapunzel proteins, which have been shown to play a role in the zebrafish immune response (Lu et al., 2019), a homolog of MRC1, a C-type lectin implicated in pathogen phagocytosis and antigen presentation in human macrophages (Miller et al., 2008; Op Den Brouw et al., 2009), several ribosomal proteins and large fraction of unannotated proteins (Fig. 2D). For further experiments, we excluded genes with housekeeping functions (e.g. ribosomal proteins) and cytoskeletal proteins from the biotinylated DNA IP results. Overall, we selected ten genes from our homology search for potential transgene expression antagonists (two *DNase2a* genes, two *dicer* genes, *piwi-1*, *TBK1*, *IRF3/4*, *TLR9*, *ATG7* and *ATG9*) and 19 genes from the list of potential transfected DNA interactors (Fig. 2E) for functional analysis.

To screen for potential transgene expression suppression effects, we performed RNAi-mediated knockdowns of each gene prior to transfection with the *eF1a-NanoLuc* reporter plasmid. NanoLuc expression was analyzed at 3 days after transfection, i.e. at the expression peak of reporter signal in wild-type worms (Fig. 1E). Most of the RNAi conditions did not affect the baseline NanoLuc signal. Intriguingly, however, RNAi of two genes enhanced the reporter signal: *TBK1(RNAi)* robustly increased average NanoLuc expression by 3.2-fold and *MRC1(RNAi)* 1.8-fold (Fig. 2E), thus indicating that the function of these genes in *Smed* suppresses reporter expression. RNAi mediated knockdown followed by NanoLuc reporter transfection was repeated for these two genes with different concentrations of dsRNA, different numbers of dsRNA feedings and with simultaneous knockdown of both genes (Fig. 2F). The average increase of NanoLuc expression over control RNAi worms [*eGFP(RNAi)*] was similar across all tested conditions, including the double knockdown of *TBK1* and *MRC1* (Fig. 2F). Even though individual worms displayed up to 10-fold [*TBK1(RNAi)*] and 5-fold [*MRC1(RNAi)*] increases in luciferase signal over *eGFP(RNAi)* controls (Fig. 2F), the boost in reporter signal was still insufficient for the robust detection of fluorescent reporters (not shown). Nevertheless, these data demonstrate that MRC1 and TBK1 limit transgene expression in planarians, thus confirming the existence of endogenous barriers to transgene expression.

### TBK1 regulates a transcriptional response to wounding

In order to gain insight into the nature of the barrier, we next focused on characterizing planarian TBK1 as the so far strongest suppressor of NanoLuc expression. *Smed* TBK1 (hereafter referred to as TBK1) is encoded by the transcript dd_Smed_v6_1972_0_1, which is expressed throughout the organism according to single-cell RNA-sequencing data (Fincher et al., 2018; Plass et al., 2018). However, whole-mount fluorescence *in situ* hybridization (FISH) staining suggested an enriched expression in intestinal cells (Fig. S2A). TBK1 displays high sequence similarity to human TBK1 but also to human inhibitor of kappaB kinase epsilon (IKKε; Fig. S3). In mammals, TBK1 and IKKε have largely redundant functions in activating IRF3 and NF-κB transcription factors during the innate immune response and in response to various other stress signals (Balka et al., 2020; Al Hamrashdi and Brady, 2022). Many upstream signaling cascades converge on these central, highly conserved kinases (Zhou et al., 2020). *TBK1(RNAi)* caused no overt phenotypes in homeostatic worms or during regeneration, even after prolonged knockdown (eight dsRNA feedings; Fig. S2B), potentially consistent with the conservation of its role as a stress-activated kinase. As discussed earlier, we did not identify clear homologs of IRF3 and NF-κB in *Smed.* RNAi-mediated knockdown of the RHD-containing gene with the highest sequence similarity to *Drosophila* Relish did not change NanoLuc expression and resulted in rapid lethality (Fig. S4A,B), indicating that this gene is not involved in the suppression of transgene expression.

To further elucidate the cellular mechanisms operating downstream of *Smed* TBK1 and their potential impact on transgene expression, we conducted a bulk RNA-sequencing experiment on worms following plasmid transfection.

**Fig. 3.**
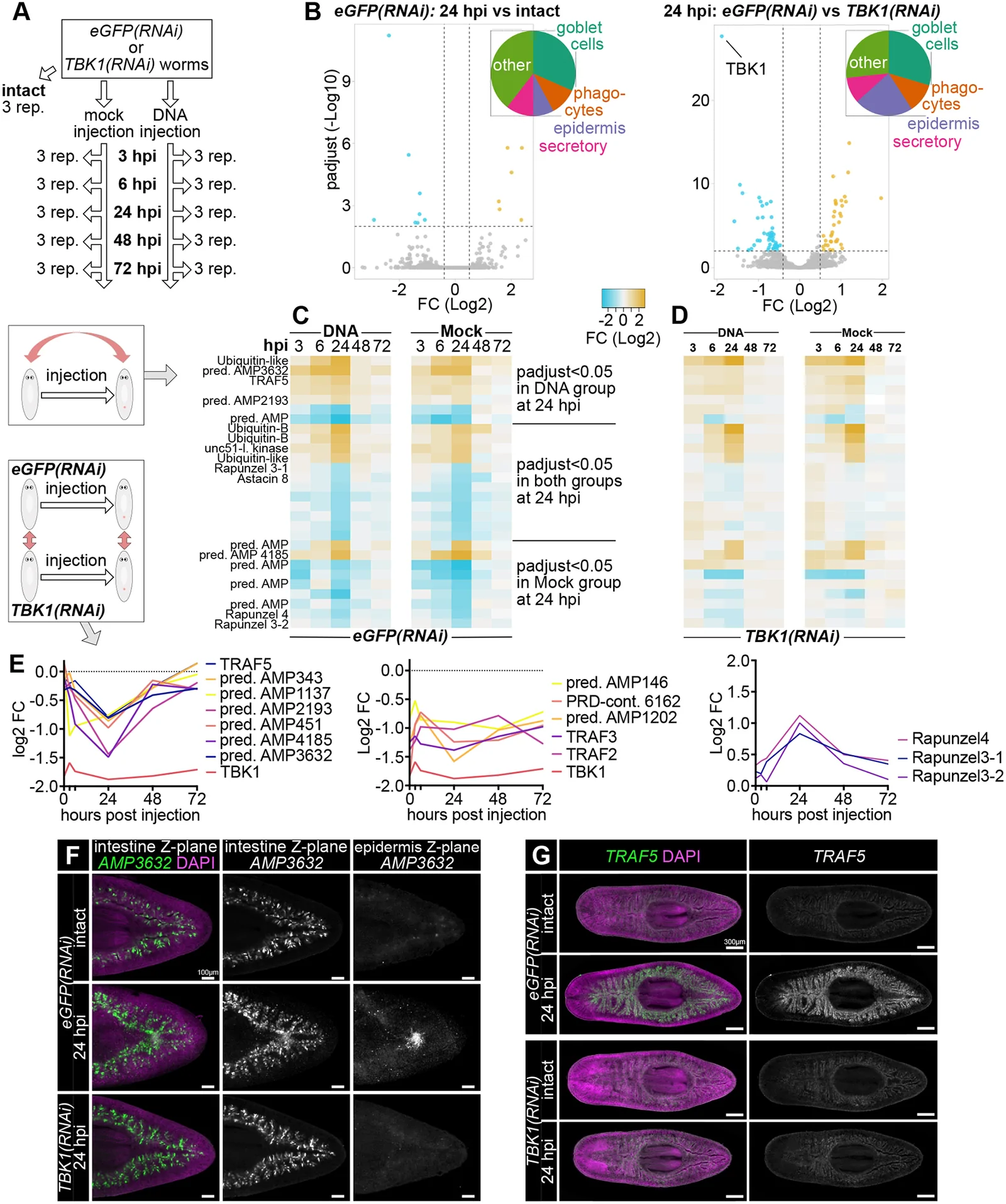
**TBK1 regulates local and global transcriptional responses to injection.** (A) Schematic experimental layout to probe for TBK1-dependent aspects of the injection response. *N*=3 biological replicates for each experimental condition. (B) Differential gene expression at 24 hpi in *eGFP(RNAi)* controls versus intact/uninjected *eGFP(RNAi)* controls (left) and *TBK1(RNAi)* versus *eGFP(RNAi)* control (right). Mock and DNA-transfected samples were combined for this analysis. Genes with *P*<0.05 are colored. The pie charts summarize the cell types in which the differentially expressed genes are enriched (see Materials and Methods). (C,D) Expression kinetics of the injection response genes [adjusted *P*<0.05 at 24 hpi in *eGFP(RNAi)*] in either *eGFP(RNAi)* (C) or *TBK1(RNAi)* (D) and under mock versus DNA transfection injection. Color coding represents log2 fold change (FC) versus intact controls. Gene symbols of conserved genes or gene features are indicated; no annotations imply lack of conservation. Pred. AMP, predicted anti-microbial peptide. (E) Cross-comparison of the expression kinetics of the indicated genes from C,D between *eGFP(RNAi)* and *TBK1(RNAi*. The graphs trace the log2 FC between *TBK(RNAi)* and control at each time point. Downward deflections imply lower expression in *TBK1(RNAi)*, upward deflections increased expression in *TBK1(RNAi)*. (F,G) Representative confocal *z*-sections of whole-mount FISH staining of *AMP3632* (F) and *TRAF5* (G) expression in *eGFP(RNAi)* and *TBK1(RNAi)* worms at 24 hpi. Note that the injection-induced upregulation of *AMP3632* locally in the epidermis (F) and of *TRAF5* globally in the intestine (G) is blocked by *TBK1(RNAi)*. Nuclear DAPI labeling was used as counterstain. Scale bars: 100 µm in F; 300 µm in G.

Specifically, we collected RNA samples of control [*eGFP(RNAi)*] and *TBK1(RNAi)* worms before transfection (‘intact’) and at 3, 6, 24, 48 and 72 h post-injection (hpi) with either a Mock transfection mix (no DNA) or a transfection mix with DNA (Fig. 3A). *eGFP* controls displayed significant differentially expressed genes (DEGs) at 24 hpi compared to the pre-injection baseline (Fig. 3B; Table S5), which collectively represents the transcriptional response to the injection injury. No significantly changed expression levels were detectable when comparing time points other than 24 hpi compared with the intact baseline. Expression levels from these time points were therefore only used to reveal trends in levels of 24 hpi DEGs. Interestingly, the injection response at 24 hpi was altered in *TBK1(RNAi)* worms, resulting in a set of DEGs relative to 24 hpi *eGFP(RNAi)* worms, including the downregulation of *TBK1* itself (Fig. 3B; Table S5).

Plotting the expression levels of injection-response genes (DEGs at 24 hpi) from the *eGFP(RNAi)* group across time points after injection showed that their expression returned to baseline by 72 hpi (Fig. 3C). These genes also behaved similarly in *TBK1(RNAi)* worms, also returning to baseline by 72 hpi (Fig. 3C,D); however, in contrast to *eGFP(RNAi)*, many of these genes did not significantly diverge from baseline at 24 hpi in *TBK1(RNAi)* worms (Fig. 3C,D).

However, we could not detect significant gene expression differences between mock and DNA transfections (Fig. 3C). The few apparent expression differences at 24 hpi between DNA transfection or Mock groups also displayed very similar expression dynamics and amplitudes in the other group, but failed to reach statistical significance (Fig. 3C). Therefore, the injection response in our data set is likely dominated by the combined effects of the injection injury and liquid injection, but the cell-autonomous effects of transfection in the likely few transfected cells may not be recovered by our whole-animal sequencing strategy. Irrespectively, the significantly reduced changes in gene expression after injection in *TBK1(RNAi)* worms compared to *eGFP(RNAi)* worms reveal a clear role of TBK1 in coordination of the transcriptional response to injection (Fig. 3D). Specifically, the *TBK1(RNAi)*-responsive genes cluster into three groups: (1) genes that are upregulated after injection in the control group but not in *TBK1(RNAi)* (reduced expression at 24 hpi in *TBK1* knockdown worms; Fig. 3E, first graph); (2) genes that are constitutively expressed at lower levels in *TBK1(RNAi)* worms at all time points (Fig. 3E, second graph); (3) genes that are downregulated in control worms at 24 hpi, but not in *TBK1(RNAi)* (Fig. 3E, third graph).

To determine the cells and tissues in which TBK1 exerts its regulatory function, we next analyzed the expression patterns of several of the injection response components by whole-mount FISH (Fig. 3F,G; Fig. S5A,B) and by querying the *Smed* single-cell RNAseq atlases (Fincher et al., 2018; Plass et al., 2018). Interestingly, most of the TBK1-dependent injection response genes were specifically expressed or highly enriched in either one of the intestinal cell types (goblet cells and phagocytes) or the epidermis (Fig. 3B,F,G; Fig. S5A,B). Both are barrier epithelia that are known to mount the first line of defense innate immunity responses in many animals (Günther and Seyfert, 2018; Rathinam et al., 2024), including planaria (Abnave et al., 2014; Kangale et al., 2021). To probe the spatiotemporal dynamics of the injection response, we carried out whole-mount FISH staining at different time points post injection. Interestingly, these experiments revealed two types of gene expression responses to injection: some genes, including *AMP3632*, were only upregulated in close proximity to the injection wound at 24 hpi (Fig. 3F); others, including *TRAF5*, were globally induced in the gut at 24 hpi (Fig. 3G). Both local and global changes in expression were clearly dependent on *TBK1*, as they were both strongly reduced in *TBK1(RNAi)* worms (Fig. 3F,G). Similarly, *TBK1* was also required for the induction of *AMP3632* (at the wound site) and *TRAF5* (throughout the intestine) expression after amputation (Fig. S5C). TBK1-dependent injection/wounding responses therefore include local effects in the immediate wound vicinity, but also global gene expression changes in the animal, particularly within the barrier epithelia of the epidermis and the intestine.

### TBK1 regulates expression of innate immune response genes

**Fig. 4.**
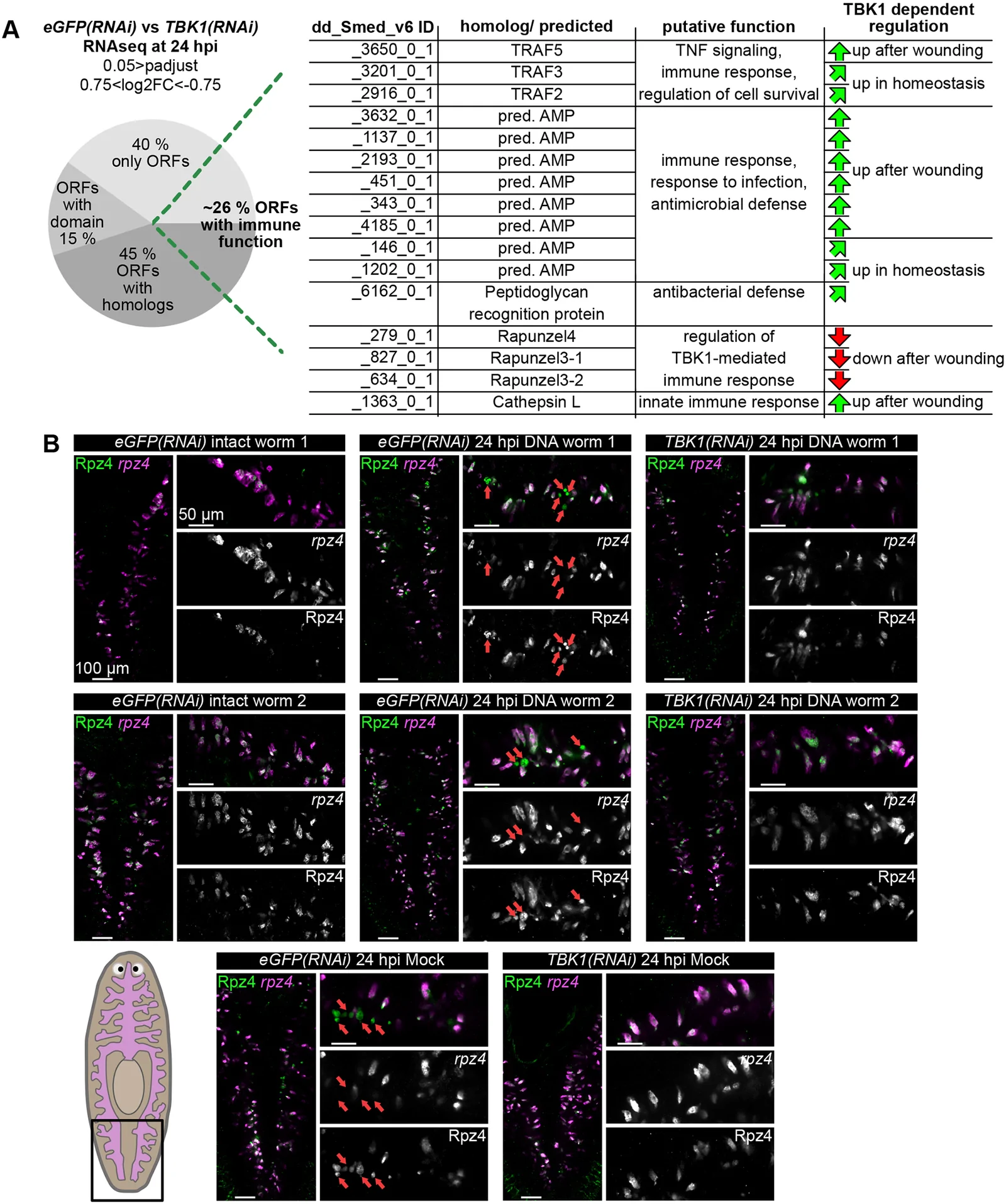
**TBK1 regulates the transcription of genes with links to innate immunity and Rapunzel protein localization.** (A) Pie chart of the 60 differentially expressed genes between *TBK1(RNAi)* and *eGFP(RNAi)* at 24 hpi (*P*adjust<0.05, 0.75<log2FC>−0.75) showing the fraction of encoded proteins without annotated domain or homology (only ORFs), with annotated protein domains and with functionally annotated homologs in other species; 26% of these genes encode for proteins with assigned immune functions based on homology or sequence composition listed below. Assigned putative functions are based on knowledge about respective homologs in other animals of TRAFs (Ha et al., 2009), AMPs (Lai and Gallo, 2009), peptidoglycan recognition proteins (Dziarski and Gupta, 2006), Rapunzels (Lu et al., 2019) and Cathepsin L (Sun et al., 2021). pred. AMP, predicted AMP. (B) Confocal *z*-sections of whole-mount specimens, co-labeled for *rpz4* transcript expression (FISH) and immunostaining of RPZ4 protein. RNAi conditions and injection status as indicated. Representative images from two independent experiments (top row, bottom row) are shown to illustrate response consistency within *eGFP(RNAi)* intact, *eGFP(RNAi)* 24 hpi with DNA and *TBK1(RNAi)* 24 hpi with DNA. Red arrows indicate instances of RPZ4 protein staining outside of *rpz4* transcript-expressing cells. Schematic shows the approximate regio shown in the images. Scale bars: 100 μm.

To investigate the possible functions of the TBK1-dependent injection response, we next analyzed the injection response gene sequences by systematically annotating ORF length, domain composition and sequence homology to functionally characterized/annotated genes in other model organisms. About 26% of the genes (16 out of 60) that we found to be regulated by TBK1 are linked to functions in innate immunity based on sequence homology or functional prediction (Fig. 4A). We identified TRAF2, -3 and -5 homologs, which are conserved signal transducers during the innate immune response (Ha et al., 2009) and a peptidoglycan-recognition domain-containing protein (dd_Smed_v6_6162_0_1) that likely has a role in defense against bacteria (Dziarski and Gupta, 2006) (Fig. 3A). Upon further inspection of the TBK1-regulated gene products, we noticed a set of ten proteins that all share a comparatively short ORF (∼100-250 amino acids), a predicted N-terminal signal peptide and contain repetitive patterns of charged amino acid sequences (Fig. S6). These are all hallmarks of antimicrobial peptide (AMP) precursor proteins (Ahmed and Hammami, 2019; Koehbach and Craik, 2019). Concordantly, eight out of the ten sequences achieved very high probability scores (>0.8; Fig. S6) using the AMP prediction pipeline AMP scanner vr2 (Veltri et al., 2018). The TBK-1 responsive gene set therefore likely includes a number of secreted AMPs, which are a common component of the innate immune responses to pathogen infections in multiple systems (Lai and Gallo, 2009; Tauszig et al., 2000).

A further interesting feature of the TBK1-reponsive genes were the four Rapunzel homologs that were all downregulated in response to injection (Figs 3D, 4A) and were also identified as potential interactors of transfected DNA (Fig. 2C,D). Intriguingly, the zebrafish homolog of Rapunzel-5 has been shown to directly interact with zebrafish TBK1 and to act as a negative feedback regulator of the anti-viral response by suppressing TBK1-mediated interferon production (Lu et al., 2019). The *Smed* genome encodes seven Rapunzel homologs that all have predicted N-terminal signal peptides and/or transmembrane domains (Fig. S7). *Smed* Rapunzel genes are known to be expressed in intestinal goblet cells (Forsthoefel et al., 2020; Reuter et al., 2015) but their function remains unknown.

To probe the role of Rapunzels in the *Smed* injection response, we performed whole-mount FISH staining of *rapunzel-4* (*rpz4*) together with an anti-Rapunzel-4 (RPZ4) antibody (Reuter et al., 2015) to visualize transcript and protein expression simultaneously. In uninjected controls, both labels colocalized in intestinal cells with the characteristic shape and distribution of goblet cells, as described previously (Reuter et al., 2015), but with varying staining intensities between individuals (Fig. 4B). However, 24 h after injection with either a plasmid transfection mix or a mock transfection mix lacking DNA, the RPZ4 antibody additionally labeled small, globular structures near *rpz4*-expressing cells, although these structures were negative for *rpz4* mRNA (Fig. 4B). Interestingly, the appearance of these RPZ4^+^/*rpz4*^−^ structures depended on TBK1, as only the double-positive cells were observed in *TBK1(RNAi)* worms (Fig. 4B). These data support a functional link between TBK1 and RPZ4 also in planarians (see below; Lu et al., 2019). Overall, the complement of TBK1-regulated genes clearly indicates that planarian TBK1 functions as a regulatory hub in innate immunity signaling.

### TBK1 responds to various immune system stimulations

**Fig. 5.**
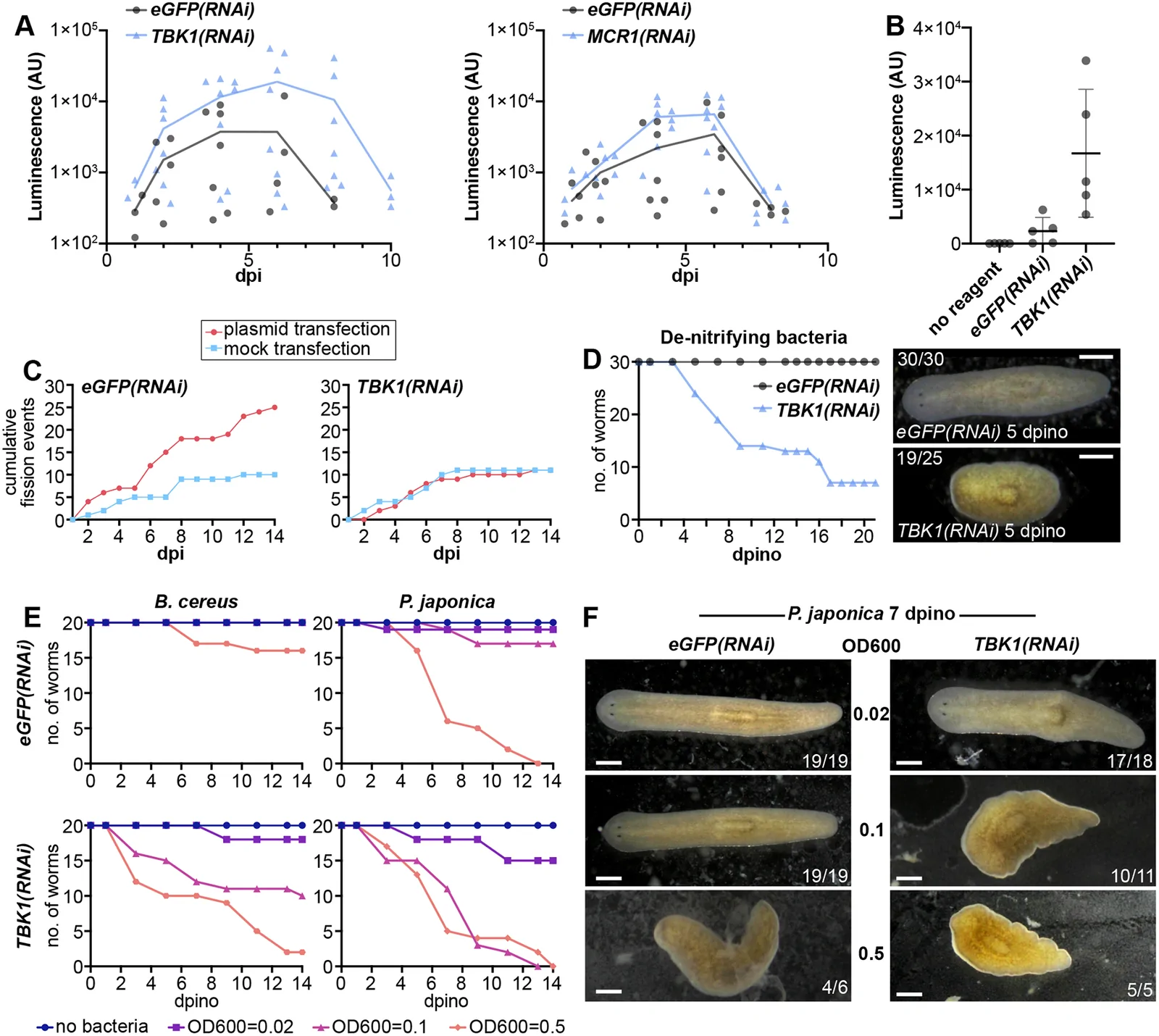
**TBK1 has a broad role in the response to foreign nucleic acids, pathogens and stress.** (A) Time course of NanoLuc expression in *TBK1(RNAi)* and *MRC1(RNAi)* worms after injection/transfection with NanoLuc reporter plasmid in comparison to control groups [*eGFP(RNAi)*]. Only positive worms are included (NanoLuc signal>mean background of untransfected controls). *N*=20 worms/time point and condition. Circles represent values for single worms and lines connect the mean value at each time point. (B) *TBK1(RNAi)* also enhances mRNA-based reporter expression. *eGFP(RNAi)* and *TBK1(RNAi)* worms were injected/transfected with NanoLuc-encoding mRNA. Data points represent the NanoLuc signal of individual worms at 1 dpi, the central bar represents the mean, and error bars represent s.d. *P*=0.0035 *eGFP(RNAi)* versus *TBK1(RNAi)* by unpaired *t*-test. (C) *TBK1* mediates the fission response to DNA transfection. Plots of cumulative fission events in response to mock or plasmid injection/transfection in *eGFP(RNAi)* (left) and *TBK1(RNAi)* (right). *N*=20 worms/condition. (D) Differential susceptibility of *TBK1(RNAi)* worms to commercial de-nitrifying bacteria. Number of surviving worms at days post-inoculation (dpino) are shown. Most surviving *TBK1(RNAi)* worms at 5 dpino show head regression (19 of 25 worms). Scale bars: 300 µm. (E) Increased susceptibility of *TBK1(RNAi)* worms (bottom) compared to *eGFP(RNAi)* controls (top) to inoculation with *Bacillus cereus* (left) or *Pseudomonas japonica* (right) bacteria. Number of surviving worms at dpino with the indicated concentration of bacteria at t=0 are shown. (F) Representative images of surviving *eGFP(RNAi)* (top) and *TBK1(RNAi)* (bottom) worms at 7 dpino with the indicated concentrations of *P. japonica*. Number pairs indicate the fraction of surviving animals displaying the phenotype. Scale bars: 300 µm. AU, arbitrary units.

Given the likely conserved function of *Smed* TBK1 as a regulatory hub in innate immune signaling, we returned to the question of how it affects transgene expression. To delineate the time window during which TBK1 and MRC1 affect transgene expression, we performed time-course experiments comparing NanoLuc reporter signal dynamics after plasmid injection into control [*eGFP(RNAi)*], *TBK1(RNAi)* or *MRC1(RNAi)* worms. In *TBK1(RNAi)* worms, both mean expression levels above background and the number of expressing worms were increased at all tested time points (Fig. 5A); moreover, the signal remained detectable at 10 days post-injection (dpi), unlike in controls (Fig. 5A). In contrast, in *MRC1(RNAi)* worms the NanoLuc signal was elevated only at 4 and 6 dpi, with no worms exceeding *eGFP(RNAi)* levels at later time points (Fig. 5A). Elucidating whether TBK1 and MRC1 affect transfection efficiency, reporter stability, or survival of expressing cells will require the development of reporter constructs enabling direct visualization of transfected cells (see Discussion). Given the stronger effect of TBK1 on NanoLuc expression, we focused subsequent experiments on its roles in immunity and stress responses.

To test whether TBK1 is specifically involved in DNA sensing or responds more broadly to nucleic acids, we assayed the expression of our previously described mRNA NanoLuc reporter (Hall et al., 2022). Interestingly, *TBK1(RNAi)* enhanced the signal of the mRNA reporter to a similar extent as the DNA reporter (Fig. 5B), suggesting a broad role for TBK1 in general nucleic acid sensing in *Smed*. Based on the role of TBK1 in coordinating the injection response (Figs 3 and 4), its effects on reporter expression could either be general and a non-cell-autonomous consequence of the injury response, or additionally involve direct and cell-autonomous roles in nucleic acid sensing. We therefore examined the dependence on TBK1 of the DNA transfection-dependent fission response, which is not triggered by the injection injury alone (Fig. 1F). Our finding that *TBK1(RNAi)* blocked the transfection-dependent stimulation of fission (Fig. 5C) therefore indicates a role of TBK1 in the sensing of transfected nucleic acids and/or in the coordination of the global response downstream. Interestingly, we serendipitously found that fission was also significantly stimulated by lethal irradiation (6000 RAD) and that *TBK1(RNAi)* similarly blocked this response (Fig. S8), possibly indicating that DNA release during irradiation-induced cell death (Pellettieri et al., 2010) may also activate the pathway. Overall, our results show that Smed-TBK1 couples nucleic acid sensing to physiological responses, reminiscent of its roles in anti-viral immunity in other systems (Zhou et al., 2020).

Vertebrate TBK1 is activated by multiple upstream pattern-sensing pathways, not only by nucleic acids. To determine whether *Smed* TBK1 also functions in other branches of innate immunity, we examined its role in planarian responses to bacterial challenges. To explore the involvement of *Smed* TBK1 in other branches of innate immunity, we assayed its role during bacterial challenges. Interestingly, *TBK1(RNAi)* worms were highly sensitive to commercial aquarium starter cultures of de-nitrifying bacteria (commonly used as beneficial supplements for planaria). In sharp contrast to the *eGFP(RNAi)* controls, which were entirely unaffected by the addition of starter cultures, *TBK1(RNAi)* worms developed head regression and significant lethality (Fig. 5D). Because these results could also reflect the response to an unknown product additive, we next examined the role of TBK1 in the defense against a genuine planarian pathogen. Since few planarian pathogens have been identified (Arnold et al., 2016), we isolated bacteria from antibiotic-free worm cultures by streaking water and tissue samples on LB agar plates and inferred species identity of the forming colonies by 16s rRNA sequencing (see Materials and Methods; Table S4). Initial screens with the addition of increasing bacterial concentrations to *Smed* planarian water identified *Pseudomonas japonica* and *Bacillus cereus* as potent inducers of lethality beyond a low threshold concentration. Titrating a range of concentrations of these two putative pathogens into the cultures again revealed strongly enhanced sensitivity of *TBK1(RNAi)* worms to both bacterial species, manifesting in head regression and lethality at concentrations in which *eGFP(RNAi)* controls were entirely unaffected (Fig. 5E,F). Taken together, our results identify TBK1 as a central hub in planarian innate immunity signaling, functions of which include the sensing of nucleic acids and the suppression of transgene expression.

## DISCUSSION

**Fig. 6.**
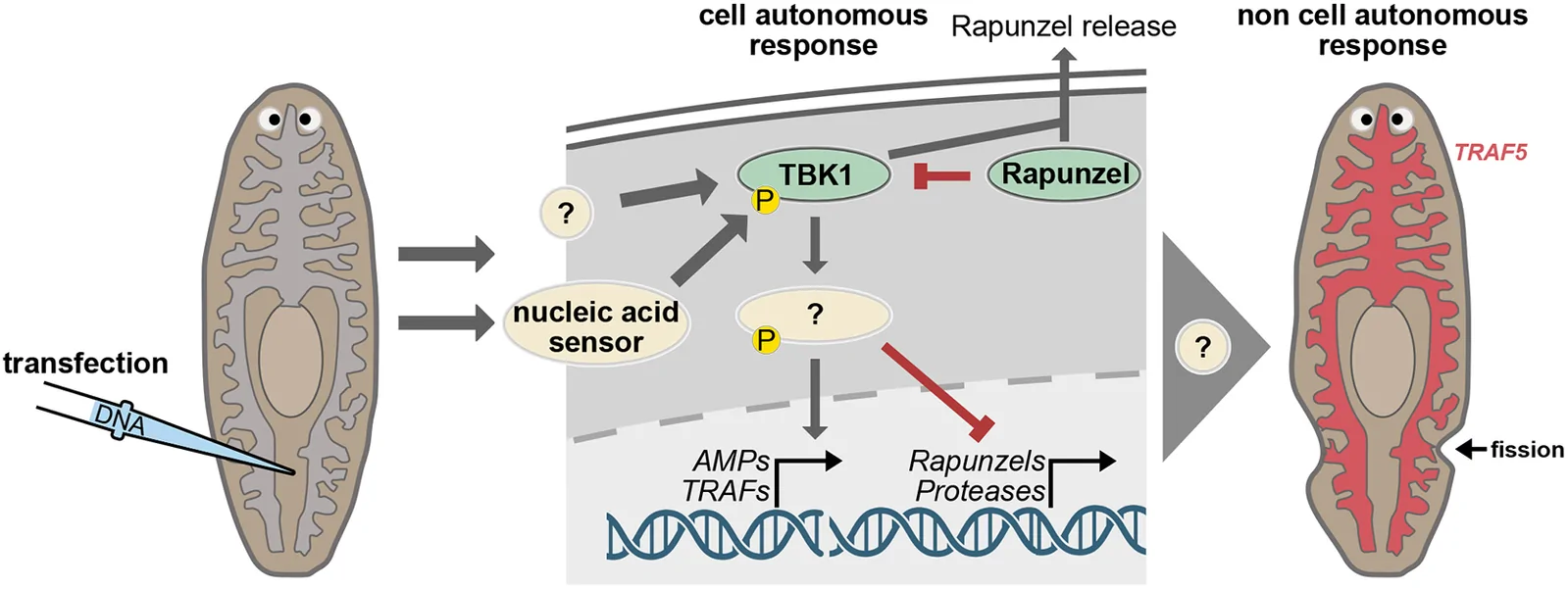
**Proposed model of TBK1 function in *Smed.*** Injection of a DNA-containing transfection mix activates TBK1 via a general wound response program and likely via nucleic acid sensors. Upon activation, TBK1 induces a transcriptional response and antagonizes its own inhibition by promoting release of Rapunzel proteins.

In this study, we establish proof of principle of plasmid-based transgene expression in *Smed* and show that innate immunity components limit transgene expression. Specifically, we show that the *Smed* homolog of TBK1 restricts the level and the duration of transgene expression, while also mediating transcriptional and physiological responses to foreign nucleic acids, bacteria and wounding (Fig. 6). The transcriptional response encompasses both local and global changes in gene expression, including the upregulation of *TRAF* and *AMP* gene expression and downregulation of Rapunzel and protease-encoding genes (Fig. 6). Given that a large fraction of the TBK1-regulated genes have likely immune-related functions and that TBK1 is required for the defense against bacteria (Fig. 4A), our results indicate deep conservation of the role of as a central hub in innate immunity signaling (Zhou et al., 2020). Our demonstration that transgene transfection triggers fission in a TBK1-dependent manner further suggests that fission may not only be an asexual reproduction strategy in planarians, but also a potential pathogen defense strategy. Conceptually, the physical shedding of compromised body parts in a whole-body regeneration-competent organism provides an effective means of physically containing infections and increasing the survival probability of the individual and the population.

One of the key questions raised by our study is the identity of the nucleic acid-sensing mechanisms upstream of TBK1. In vertebrates and insects, TBK1 functions downstream of the cGAS/STING pathway (Zhou et al., 2020; Decout et al., 2021). Surprisingly, we were unable to identify homologs of either cGAS or STING in *Smed*, which is unexpected given the bacterial origins and broad phylogenetic conservation of this pathway (Patel et al., 2023). Similarly, we failed to identify clear homologs of the other currently known DNA sensors (e.g. Aim2, TLRs; Fig. 2A). Therefore, the TBK1-dependent suppression of transgene expression and the TBK1-mediated, transfection-induced fission response suggest that planarians rely on yet-uncharacterized DNA sensors upstream of TBK1. To explore these pathways, we performed mass spectrometry on DNA pull-downs and identified a planarian homolog of MRC1, which modestly increased transgene expression upon knockdown (Fig. 2B-F). Although MRC1 has been implicated in innate immunity in vertebrates, the modest effect observed in *MRC1(RNAi)* animals, along with the lack of synergy in TBK1/MRC1 double knockdowns, argues against MRC1 as the major DNA sensor upstream of TBK1. In addition, we identified several Rapunzel homologs among the putative DNA-binding proteins. In zebrafish, Rapunzel 5 has been shown to directly interact with TBK1 and inhibit its antiviral activity (Lu et al., 2019). Although direct protein–protein interaction assays were beyond the scope of this study, our observation of TBK1-dependent transcriptional downregulation of *rpz* genes and TBK1-dependent changes in Rpz protein localization following injection nevertheless establish strong functional links between TBK1 and Rapunzel in planarians. While knockdown of individual Rpz homologs did not affect transgene expression (not shown), a role in nucleic acid sensing remains plausible due to potential redundancy among the seven *rpz* genes (Fig. S7), which cannot currently be targeted simultaneously by RNAi in *Smed*. Moreover, the observed transcriptional downregulation of *rpz* genes and the detection of Rpz4 protein outside of *rpz*-expressing cells (possibly reflecting secretion) support the idea that Rpz proteins may act as negative-feedback regulators of TBK1. This regulation could help relieve constitutive TBK1 inhibition, thereby enabling activation of downstream immune responses (Fig. 6). Whether Rpz proteins in planarians and other animals contribute directly to DNA sensing, and, more broadly, how DNA sensing upstream of TBK1 occurs in planarians, are some of the interesting avenues for future investigation.

### Cell autonomous and non-cell-autonomous immune response mechanisms

A second major question raised by our findings concerns the mechanisms that coordinate transcriptional responses downstream of TBK1. In mammals, the principal targets of TBK1 are the RHD transcription factor paralogs NF-κB and IRF3, which activate interferon (IFN) expression upon phosphorylation and activation by TBK1, respectively (Zhou et al., 2020). Although seven RHD transcription factors are present in the *Smed* genome, they are highly divergent from other invertebrate NF-κB homologs in phylogenetic trees, evident by long branches (Fig. S4C). Moreover, the highest confidence Smed Class-I NF-κB homolog (SMEST009320002.1) and Smed IRF3 homolog did not influence transgene expression, suggesting TBK1-independent functions (Fig. 2E; Fig. S4B). However, the TBK1-dependent upregulation of injection response genes in the epidermis and intestine (Fig. 3B,F,G) demonstrates that planarian TBK1 does mediate transcriptional changes, likely through a different family of transcription factors. Interestingly, the combination of local (injection site vicinity) and systemic (throughout the gut) gene expression changes that we observed (Fig. 3F,G) is generally a hallmark of innate immune signaling downstream of TBK1. In mammals, NF-κB and IRF targets include IFNs, a class of cytokines that are secreted in response to various stimuli (infection, stress, cell death). This enables local cell-autonomous immune sensing to generate a tissue-scale or even system-wide response (Zhou et al., 2020; Wang et al., 2024). Although IFNs are absent in invertebrates, it is tempting to speculate that planarian TBK1 may similarly trigger the transcription of long-range signals in the cells where it becomes activated, e.g. by transfected DNA. Accordingly, the group of early, locally induced injection response genes including AMP3632 in our data (Fig. 3C,D) might include planarian immune signals. Intriguingly, in multiple systems, AMPs have been shown to modulate immune responses and gene expression patterns via cell surface receptor binding (Lai and Gallo, 2009; Lee et al., 2019). Hence, AMP3632 and the other AMP-like molecules are promising candidate mediators of long-range ‘danger’ signaling in *Smed*, downstream of TBK1.

### Towards transgenic tools in planaria

The most intriguing implication of our findings is that endogenous innate immunity pathways limit transgene expression in planarians. The overexpression of exogenous genes, and transgenesis in general, has proven vexingly difficult in the planarian model species *Smed* and remains a major methodological bottleneck in the field. In this context, our proof-of-principle demonstration of transient plasmid-based expression of a NanoLuc reporter represents a significant step forward. The plasmid series we provide, incorporating a range of endogenous regulatory sequences from ubiquitously expressed and neoblast-specific genes, provides a useful resource for further methodological optimization by the community. Our observation of NanoLuc signals with constructs incorporating putative regulatory regions of both universally and neoblast-specifically expressed genes, but not differentiated cell-specific genes (Fig. 1C), is consistent with expression within pluripotent neoblasts. The absence of a NanoLuc signal in lethally irradiated and stem cell-depleted animals provides further evidence for this (Fig. 1D). The optimization of the currently rather low expression levels is certainly an important objective. Although our current protocol (Figs 1 and 5B) and previous mRNA reporters yield unambiguous NanoLuc signals orders of magnitude above background, the number of reporter proteins per cell remains below the detection limit of current fluorescent reporters (not shown). Our transfection protocol and reporter plasmids now provide an opportunity to start exploring the various contributions of translation initiation sequence motifs, 5′ and 3′ UTR sequences or splicing to the optimization of reporter expression levels.

Our finding that TBK1(RNAi) boosts NanoLuc reporter activity up to tenfold in individual animals demonstrates the existence of endogenous barriers to transgenesis and provides at least a part of the answer of why transgene expression remains so challenging in planarians. A key next question is to determine how TBK1 limits transgene expression. We show that the knockdown of TBK1 increases both the level and duration of NanoLuc expression from plasmid (Fig. 5A) and the expression level from mRNA (Fig. 5B). In light of the known cell-autonomous and non-autonomous signaling roles of TBK1 in other animals, these effects might reflect the autophagic degradation of transfected DNA, the inhibition of translation by anti-viral responses, or induction of apoptotic cell death and consequent removal of transfected cells (Balka et al., 2020; Liyana and Vanessa, 2019; Zhang et al., 2021). In general, fluorescent reporters and live imaging will likely be instrumental in delineating the precise mechanisms by which planarian TBK1 inhibits transgene expression. In addition, the identification and characterization of the upstream nucleic acid sensors and downstream response components promise further opportunities for boosting transgene expression levels. Innate immune sensors, including the dsDNA sensors AIM2 and cGAS, typically trigger multiple and partially redundant pathways (Han et al., 2025; Hu et al., 2016; Ablasser et al., 2014), which could be reflected in the apparently TBK1-independent components of the injection response in our RNAseq data (Fig. 3C,D). It is therefore conceivable that RNAi targeting of the most upstream sensors of the immune response and/or of key target mechanisms might offer the greatest and most specific enhancement of transgene expression levels and duration. This makes identifying the relevant genes and pathways all the more appealing.

Overall, our study initiates an experimental approach towards planarian transgenesis that combines the temporary RNAi-mediated inhibition of DNA-sensing mechanisms with further reporter plasmid optimization. In addition, it highlights the intrinsic interest and importance of understanding the unique innate immunity pathways of planarians and other invertebrates as a pertinent area for future research.

## MATERIALS AND METHODS

### Planaria laboratory cultures

The asexual CIW4 strain of *Smed* was used exclusively in this study and kept in planarian water (1.6 mM NaCl, 1 mM CaCl_2_, 1 mM MgSO_4_, 0.1 mM MgCl_2_, 0.1 mM KCl, 1.2 mM NaHCO_3_) supplemented with 50 µg/ml gentamycin sulfate in incubators at 20°C in the dark. During standard maintenance, animals were fed once a week with organic calf liver paste as described previously (Merryman et al., 2018). For regeneration assays, animals were starved for at least 1 week before amputation and washed with fresh planarian water 1 day and 5 days after amputation.

### Cloning of planarian expression plasmids

Regions of 800-2000 bp upstream and downstream of ORF coding exons of indicated genes were selected based on the *Smed* genome assembly (Ivanković et al., 2024) and amplified by PCR. A plasmid backbone based on pBluescriptII SK (+) and a *Smed* codon optimized NanoLuc-encoding ORF were amplified by PCR, including overhangs to regulatory regions, and plasmids were assembled using NEBuilder Hifi DNA assembly master mix (NEB, E2621S). Plasmid sequences were verified by Sanger sequencing of all inserts.

### Transfection of *Smed*

Plasmids for transfection were purified from overnight *Escherichia coli* cultures with the Plasmid plus Midi kit (QIAGEN, 12943), and 1 µg plasmid DNA was added to 20 µl OptiMEM medium at room temperature and briefly mixed by vortexing. TransIT-X2 transfection reagent (mirusbio, MIR 6004) was brought to room temperature before use, and 4.5 µl added to the transfection mix followed by immediate mixing by pipetting up and down six times. The transfection mix was then incubated at room temperature for 15 min before injection into planaria. The ratios of DNA and transfection reagent for other DNA transfection reagents in this study were set up according to manufacturers’ protocols using 1 µg of total plasmid DNA in 25 µl buffer.

For transfection with mRNA, 1 µg of RPL15-sNLuc2 mRNA was mixed with 0.8 µl Viromer mRNA reagent and Viromer mRNA buffer to a final volume of 25 µl and incubated for 15 min at room temperature before injection as described by Hall et al. (2022).

For injection, planaria were immobilized by placing them with the ventral side up on a moist Whatman filter paper on a cooling plate at 4°C. Next, 3.5″ glass capillaries (Drummond, 3-000-203-G/X) that were pulled using a Model P-1000 micropipette puller (Sutter Instruments) with heat=530, pull=150, velocity=150, delay=0, pressure=100, were loaded with the transfection mix after breaking the needle tip open with forceps. Injections were then performed using a FemtoJet 4i injection system (Eppendorf) with two 0.3 s pulses at 5 PSI. Worms were injected in the middle of the tailstripe region as described by Hall et al. (2022) using a separate handheld needle to pre-injure the epidermis of the worms at the desired injection site.

### Measurements of NanoLuc signal

If not indicated otherwise, NanoLuc signal was analyzed 3 days after transfection. NanoLuc signal measurements were performed as previously described (Hall et al., 2022). Briefly, single worms were homogenized using a razor blade, taken up in 225 µl Leibovitz L15 medium and transferred to a 96-well plate. Per well, 100 µl buffer and 2 µl substrate from the NanoGlo Luciferase Assay kit (Promega, N1110) were added and mixed by pipetting up and down ten times. Luminescence was then measured using a SynergyNeo2 plate reader.

### Synthesis of dsRNA and RNAi experiments

Synthesis of dsRNA and feeding of liver paste/dsRNA mix to *Smed* worms was carried out as described by Rouhana et al. (2013). Linear DNA templates for dsRNA synthesis were amplified from *pPRT4P* plasmids containing 500-1500 bp parts of the target gene cDNA using T7-AA18/PR244 primers, or from 500-1500 bp PCR products with attached T4P sequence overhangs using T7-T4PCR-for/T7-T4PCR-rev primers. Linear DNA templates were purified and then used in *in vitro* transcription reactions with T7 RNA Polymerase (Thermo Fisher Scientific, EP0111) followed by NaCl/PEG-8000 precipitation of the dsRNA. Purified dsRNA was diluted to 8 µg/µl and mixed with liver paste to a final concentration of 2 µg/µl unless indicated otherwise and stored at −80°C until feeding. Worms were fed *ad libitum* for 1 h twice a week and dishes were cleaned immediately after feeding and one day after feeding. If not otherwise indicated, worms were fed three times for experiments.

### Whole-mount FISH and immunostaining

Riboprobes were synthesized for each gene from linear DNA templates containing full length or most of the target ORF sequence and purified as described previously (Liu et al., 2013; King and Newmark, 2013). *Smed* worms were starved for 2 weeks before fixation by 5 min incubation in 5% N-acetyl-cysteine followed by fixation in 4% paraformaldehyde for 45 min at room temperature. The whole-mount FISH protocol was otherwise carried out as described previously (Liu et al., 2013; King and Newmark, 2013) and worms were subsequently mounted in Scale-S4 medium (10% glycerol, 15% DMSO, 40% sorbitol, 4 M urea, 2.5% DABCO, 0.1% Triton X-100 in H_2_O) for microscopy. The anti-RPZ4 antibody was a kind gift from Kerstin Bartscherer (MPI for Molecular Biomedicine, Münster, Germany) and used at a 1:200 dilution with anti-rabbit-Alexa Fluor 647 secondary antibody (1:100 dilution; Thermo Fisher Scientific, A-21244).

Images were acquired on an Olympus IX83fluorescence microscope with a spinning disk Yokogawa CSUW1-T2S scan head; 405 nm, 560 nm and 640 nm lasers were used with 447/50, 595/50 and 685/40 nm filter sets. A 20× (Air, NA 0.8, WD 0.6 mm) objective was used and images were recorded on a Hamamatsu Orca Flash 4.0 v3 monochrome sCMOS camera. Images were processed in Fiji (Schindelin et al., 2012).

### Total RNA extraction and RNA sequencing

Worms were starved for 2 weeks and selected for a uniform size of 6-8 mm across all samples. For total RNA extraction, three biological replicates per time point with three worms per sample were homogenized in TRIzol, incubated at room temperature for 5 min followed by adding 200 µl chloroform and vortexing. Samples were then centrifuged at 12,000 ***g*** and 4°C for 15 min and then the aqueous upper phase was transferred to a fresh tube containing 250 µl isopropanol and 250 µl high-salt solution (0.8 M sodium citrate and 1.2 M NaCl) and vortexed briefly. Samples were then further purified using the Direct-zol RNA Miniprep (Zymo Research) according to the manufacturer's instructions. RNA integrity was analyzed by agarose gel electrophoresis and samples were then diluted to 50 ng/µl. Poly-A-enriched libraries were prepared and 75 bp 3′ end sequencing was performed on the Illumina NextSeq500 platform. Obtained reads were quality checked with FastQC (v0.11.9), reads were trimmed and again checked with FastQC. Contamination was checked with Kraken2 (v2.1.1) and Centrifuge (v1.0.3) and remaining reads were then aligned to dd_Smed_v6 transcriptome using Star (v2.7.6a). Differential expression analysis was performed with deSeq2.

### Pull-down of biotinylated DNA from worm lysates

Linear DNA (mScarlet-encoding ORF) was amplified by PCR and purified followed by biotinylation of 3′ ends using Terminal Transferase (Roche, 3333566001) and biotin-d14-ATP (Jena Bioscience, NU-835-BIO14-S) according to the manufacturers’ instructions. Successful labeling was confirmed by increased size of the dsDNA, visualized by agarose gel electrophoresis. Biotin-labeled DNA and unlabeled DNA (control) were then used for transfection of worms as described above. Four transfected worms per sample were homogenized in 100 µl lysis buffer (1 M Tris/HCl, 1.5 M NaCl, 10 mM EDTA, 0.5% NP40, pH 8.0) containing 1× Halt Protease Inhibitor cocktail (ThermoFisher Scientific) 1 h after transfection and incubated for 15 min on ice followed by centrifugation at 15,000 ***g*** and 4°C for 10 min. Supernatant was moved to a fresh tube and IP of biotinylated DNA was performed using Streptavidin Magnetic Beads (NEB, S1420S) according to manufacturer instructions with a binding incubation time of 1 h at 4°C. After three washes, 1×LDS sample buffer was added to the beads for elution of bound proteins and samples were incubated at 95°C for 10 min. Five biological replicates each were prepared for samples with transfected biotinylated DNA and unlabeled transfected DNA, respectively.

### Mass spectrometry analyses

For mass spectrometry analyses, 25 μl of the DNA IP eluates were loaded on 4-12% BisTris NuPAGE SDS-PAGE gels and run in 1× MOPS running buffer at constant 125 V for 105 min. Gels were then washed three times 10 min in MilliQ H_2_O and stained with Gelcode Blue staining solution (Thermo Fisher Scientific, 24590) for 30 min. Gels were then washed in MilliQ H_2_O six times for 10 min followed by staining with Expedeon InstantBlue. The complete gel lanes were cut into 23 equally sized slices. Contained proteins were reduced with 10 mM DTT for 50 min at 56°C, alkylated with 55 mM iodoacetamide for 20 min at 25°C, and in gel digested with porcine trypsin overnight at 37°C. Resulting peptides were separated on an Acclaim PepMap100 C18 μPrecolumn (300 μm i.d.×5 mm, C18 PepMap100,100 Å, 5 μm) at a flow rate of 10 μl/min and a C18 capillary column (30 cm, 360 μm o.d., 75 μm i.d., Reprosil-Pur 120 Å, 3 μm, C18-AQ) at a flow rate of 300 nl/min, with a gradient of acetonitrile ranging from 12 to 45% in 0.1% formic acid for 54 min using an online UltiMate 3000 RSLCnano HPLC system coupled to an Orbitrap Exploris 480 mass spectrometer with FAIMS pro interface. MS conditions were as follows: compensation voltage −40 V; spray voltage, 1.8 kV; heated capillary temperature, 275°C; normalized collision energy, 30. An intensity threshold of 5.0 e3 was used. The mass spectrometer automatically switched between MS and MS/MS acquisitions (data-dependent mode). Survey MS spectra were acquired in the Orbitrap (350-1200 m/z) with the resolution set to 60,000, automatic gain control target at 3e6 injection time mode set to auto. The most intense ions of charge states 2+ to 7+ were sequentially isolated for a maximum cumulative measurement time of 1.2 s for HCD MS/MS fragmentation and detection. MS2 resolution was set to 15,000, automatic gain control target at 1e5 for a maximum of 40 ms. Dynamic exclusion was set to 30 s.

### Mass spectrometry data analysis

Raw data were analyzed with MaxQuant (v1.6.17.0) using a merged *Smed* proteome from longest ORFs for each gene of dd_Smed_v6 and dd_Smes_v2 as a sequence database. Sequences with more than 95% identity were merged. Up to two missed cleavages of trypsin were allowed. Oxidized methionine and N-terminal acetylation were searched as variable modification and cysteine carbamidomethylation as fixed modification. The false positive rate was set to 1% at the peptide level, the false discovery rate was set to 1% at the protein level, and the minimum required peptide length was set to six amino acids. The LFQ output of MaxQuant was then further analyzed with LFQ-Analyst (Shah et al., 2020) using Perseus-type imputation, Benjamini–Hochberg FDR correction and excluding single peptide identifications.

### Identification of bacteria and worm infection experiments

Lysates from worms that were kept in planarian water without antibiotics were plated on LB agar plates and incubated at 20°C for 48 h. Single colonies were subsequently amplified in liquid LB medium cultures at 20°C for 24 h at 180 rpm on a shaker. Genomic DNA was extracted and used as a template for 16s rRNA PCR using 616V and 1492R primers (Loy et al., 2002). PCR product was purified and sequenced with Sanger method (Table S4). For infection experiments, bacteria were grown in liquid LB medium at 20°C for 24 h at 180 rpm followed by centrifugation/pelleting of bacteria (11,000 ***g*** for 2 min) and resuspension in 1×PBS which was repeated twice. OD600 was measured and bacteria were then again centrifuged/pelleted (11,000 ***g*** for 2 min) and resuspended in 1× planarian water to achieve the desired OD600. The bacteria-containing water was then applied to worms that were starved for 2 weeks and refreshed every 2 days.

### Software, algorithms and repositories

Protein homologs between different species were identified using BLAST (Altschul et al., 1990). Proteins were labeled as ‘conserved in Smed’ when queries from *Drosophila* and human proteins showed highest similarity to the same planarian protein, and ‘absent in Smed’ when no Smed protein with sequence similarities was detectable. Planarian sequences, gene expression profiles and other gene parameters were obtained from Planmine (Rozanski et al., 2019). Cell type-specific expression was inferred from single-cell RNA-sequencing datasets (Plass et al., 2018) and enrichment in a specific cell type as shown in Fig. 3B was defined as mean expression levels being twice as high in one cell type, compared to the mean expression levels in other cell types. MUSCLE 5.1 (Edgar, 2004) was used for multiple sequence alignments. Geneious Prime (Dotmatics) was used for handling of sequences, cloning analysis and calculation of phylogenetic trees using its integrated Tree builder. ImageJ Fiji (Schindelin et al., 2012) was used for handling and processing of confocal microscopy images. Affinity Designer (Affinity) was used for processing of panels and generation of figures.

## Supplementary Material



10.1242/develop.205222_sup1 Supplementary information

Table S1. RNAi targeted genes and primers that were used for dsRNA synthesis template cloning. List of genes that were targeted by RNAi in this study. Transcript IDs, homologs based on sequence similarity and primer sequences that were used for cloning DNA templates for dsRNA synthesis are listed.

Table S2. Primers used for cloning of genomic regions Transcript IDs, gene names, identity of cloned regions and primers that were used for amplification of these regions are listed for genes of which regulatory regions were used to construc NanoLuc expression reporter plasmids.

Table S3. Sequence of NanoLuc codon optimized for Smed The NanoLuc encoding sequence that was codon optimized for Smed is shown.

Table S4. 16s rRNA sequences of identified bacterial species The 16s rRNA sequences that were concatenated from forward and reverse sanger sequencing of 16s rRNA-region PCR products are shown and inferred bacterial species (based on highest similarity in BLAST search) are listed.

Table S5. Differentially expressed genes at 24 hours post injection within and between *eGFP(RNAi)* and *TBK1(RNAi)* worms Gene IDs, log2 fold change, log2 fold change standard error and padjust values are listed for DEGs from indicated comparisons at 24 hpi within and between *eGFP(RNAi)* and *TBK1(RNAi)* worms. Underlying experimental layout is shown in Fig. 3A.

Table S6. Differential protein quantification of Biotin IP from worms transfected with Biotinylated DNA vs DNA Protein IDs, log2 fold change and padjust values are listed for proteins that were identified and quantified by mass spectrometry in Biotin IP samples. Samples from worms that were transfected with Biotin-labeled DNA were compared to samples from worms that were transfected with DNA (control).
