# Supplementary material for: Conserved innate immunity components limit transgene expression in adult planarians
Source: Development. 2026 Mar 27;153(6):dev205222. doi: 10.1242/dev.205222 (PMC13563047; doi:10.1242/dev.205222)
Supplement: Supplementary file 1 [file develop-153-205222-s1.pdf]

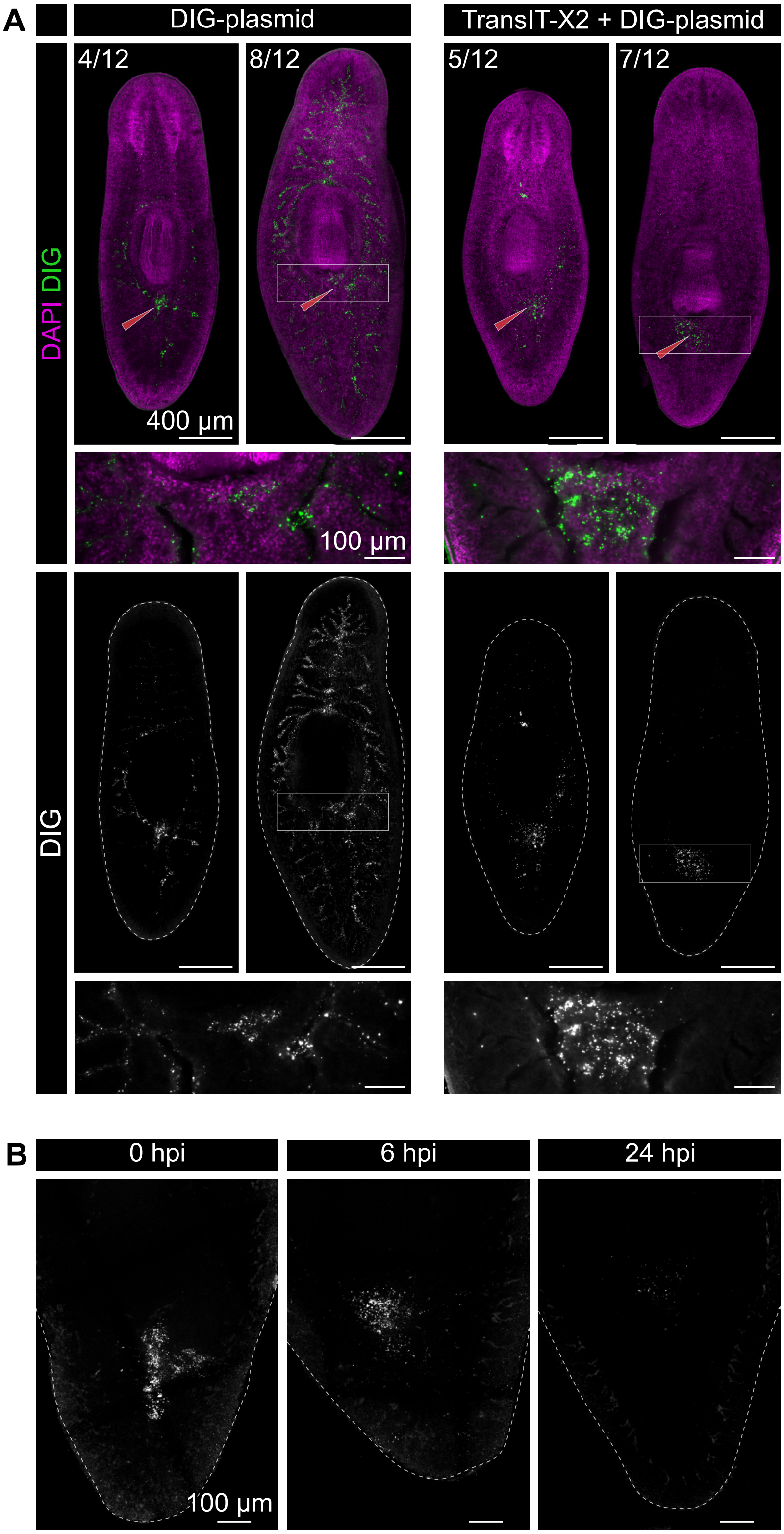

Fig. S1. Transfection reagent limits dispersion of transfected plasmid DNA

(A-B) Whole mount anti-DIG immunostainings of wildtype worms that were injected with DIG-labeled plasmid DNA together with transfection reagent ((A), right-hand panels; (B)) or without transfection reagent ((A), left-hand panels). Images are representative for the number of worms indicated in top right corners. Red trinagles indicate injection site. DIG-labeled plasmid DNA rapidly disperses into the intestine without transfection reagent (A) and is mostly degraded within 24 hpi even with transfection reagent (B). Areas of details are indicated by white boxes. Scale bars as indicated.

Development • Supplementary information

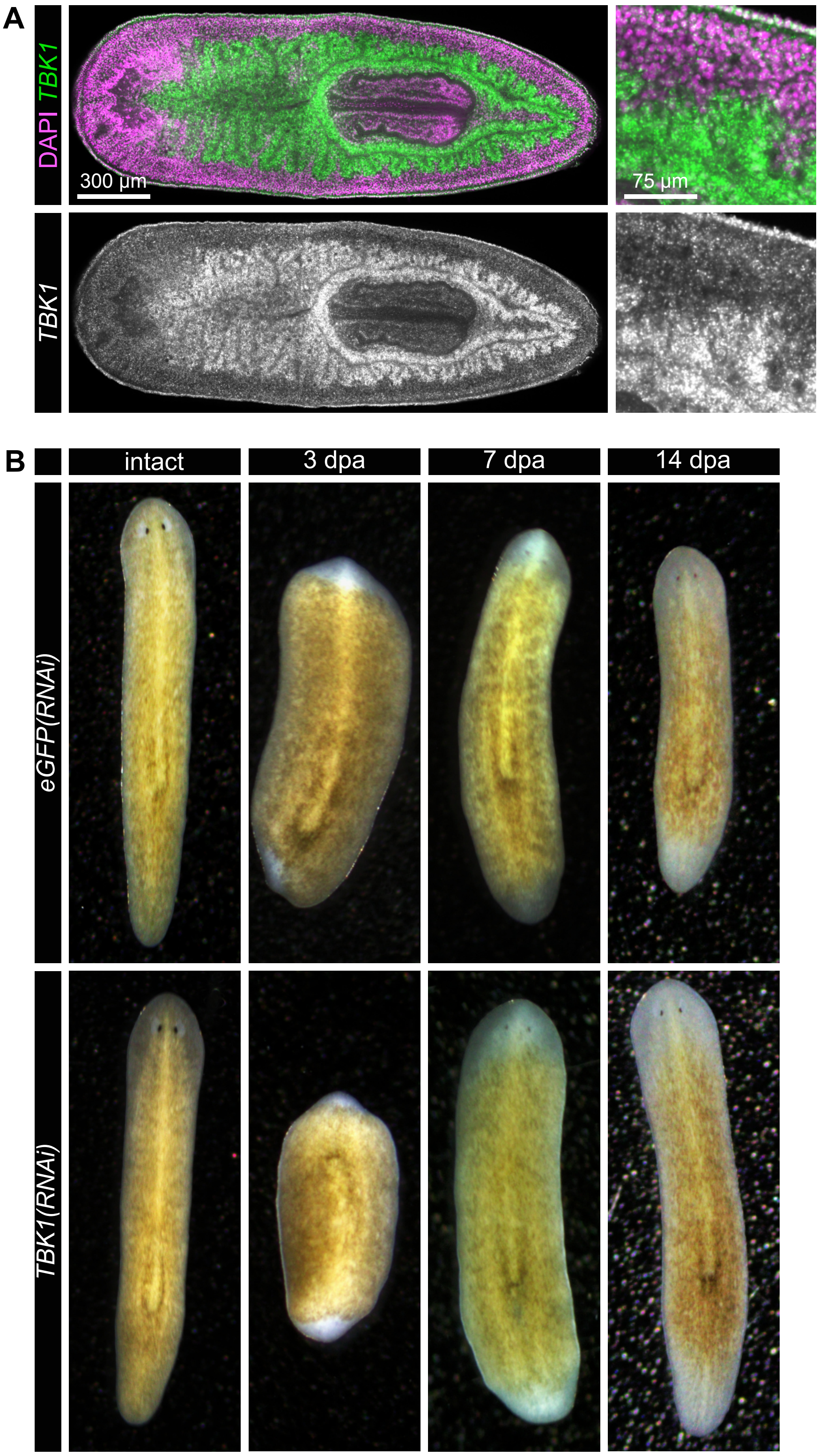

Fig. S2. *TBK1* is broadly expressed and RNAi of *TBK1* does not affect survival or regeneration
(A) Whole mount FISH staining of *TBK1* transcript in wildtype worms shows ubiquitous expression of *TBK1* with enrichment in the intestine.
(B) Intact worms and regenerating pieces at different days post amputation (dpa) of *eGFP(RNAi)* worms (top) and *TBK1(RNAi)* worms (bottom) are shown. Worms were fed 8 times with liver paste containing 2 µg/µl dsRNA and starved for two weeks before being cut into 3 pieces along the anterior/posterior axis. Images are representative for groups of 20 worms (intact) and 30 worms (regenerating pieces) respectively. All pieces, including head and tail pieces (not shown) regenerated normally.

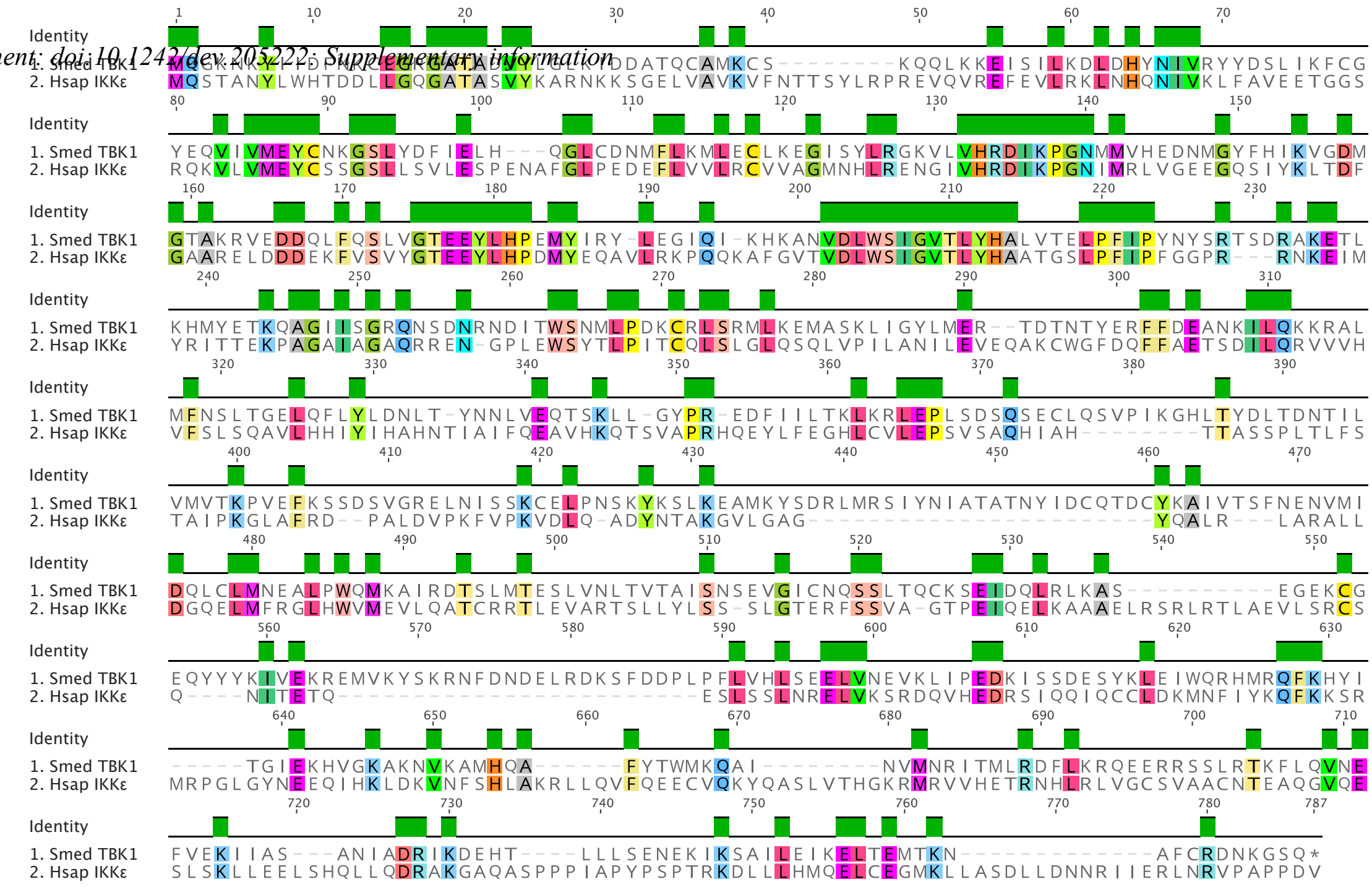

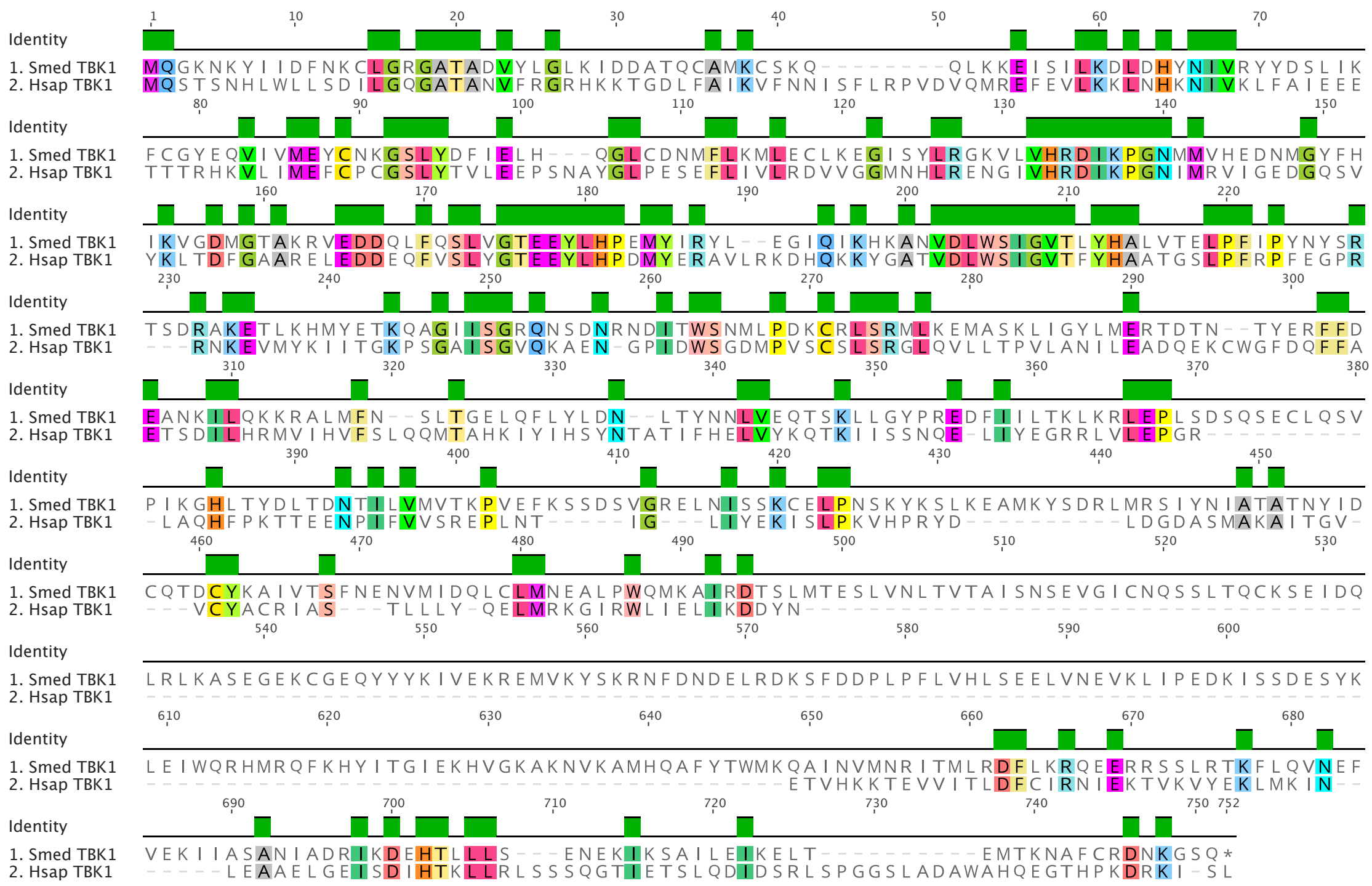

Fig. S3. *Smed* TBK1 shares homology with human TBK1 and IKKε

Sequence alignment of Smed TBK1 with human IKKε (top; GenBank: AAI05925.1) and human TBK1 (bo om; GenBank: KAI2566783.1), respec vely. Muscle 5.1 algorithm was used for alignment (Edgar 2004).

Development • Supplementary information

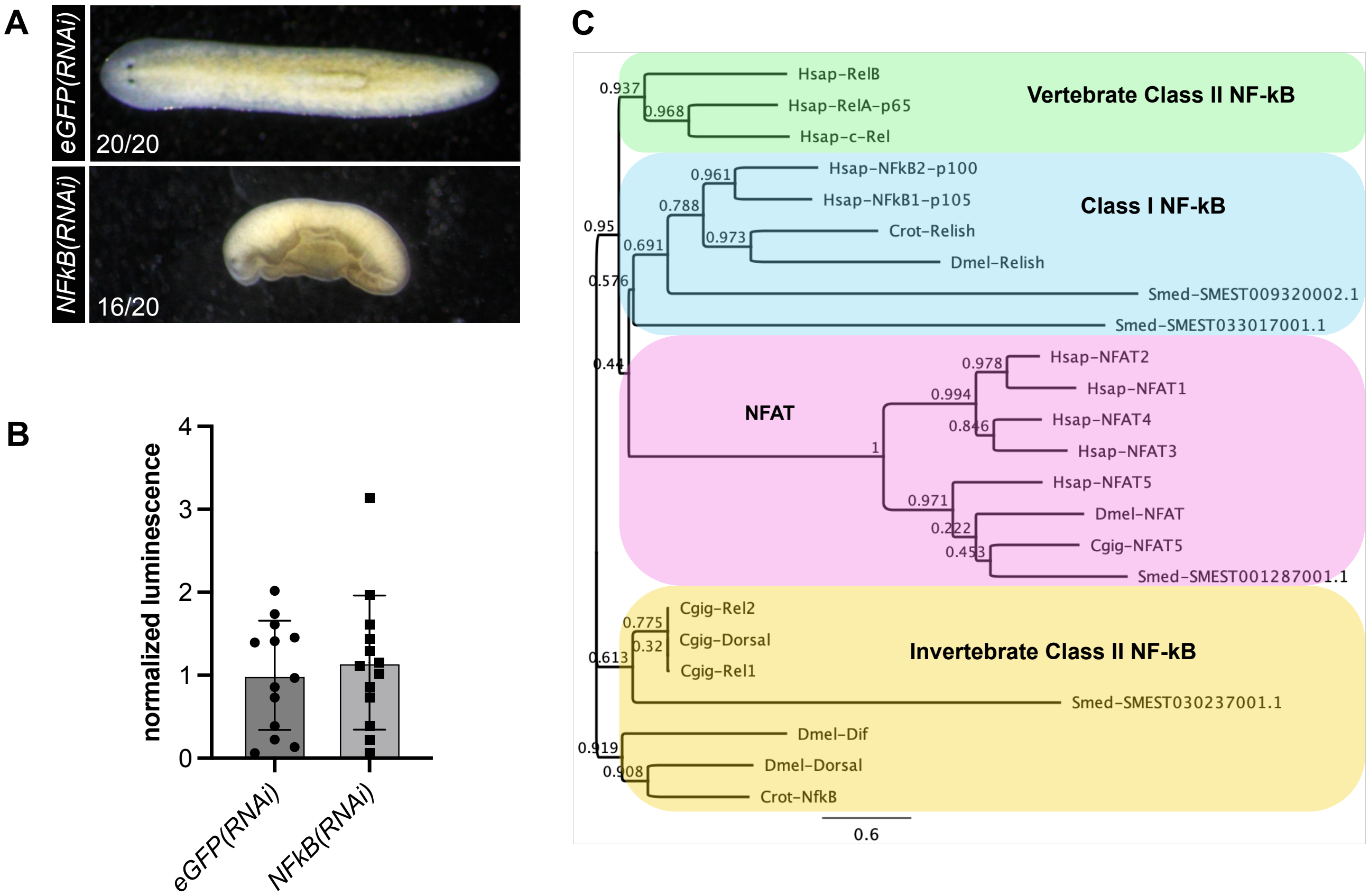

Fig. S4. RNAi of *Smed* NF-κB induces lethality but no change in NanoLuc expression levels

(A) Worms with RNAi of *eGFP* (control; top) and *NF-κB* (bottom) are shown at 5 days after a single dsRNA feeding (2 µg/µl). *NF-κB(RNAi)* worms show head regression and rapid lethality until 8 days after the dsRNA feeding.

(B) Normalized luminescence measurements for control (*eGFP(RNAi)*) and *NF-κB(RNAi)* worms taken three days after transfection with NanoLuc reporter plasmid. Each data point represents a measurement from a single worm. Worms were transfected 3 days after the dsRNA feeding.

(C) Phylogenetic tree of NF-κB transcription factors from human, Smed, *Drosophila melanogaster, Crassostrea gigas* and *Carcinoscorpius rotundicauda.* Smed NF-κB sequences show high divergence indicated by long branches with the exception of Smed NFAT5 homolog.

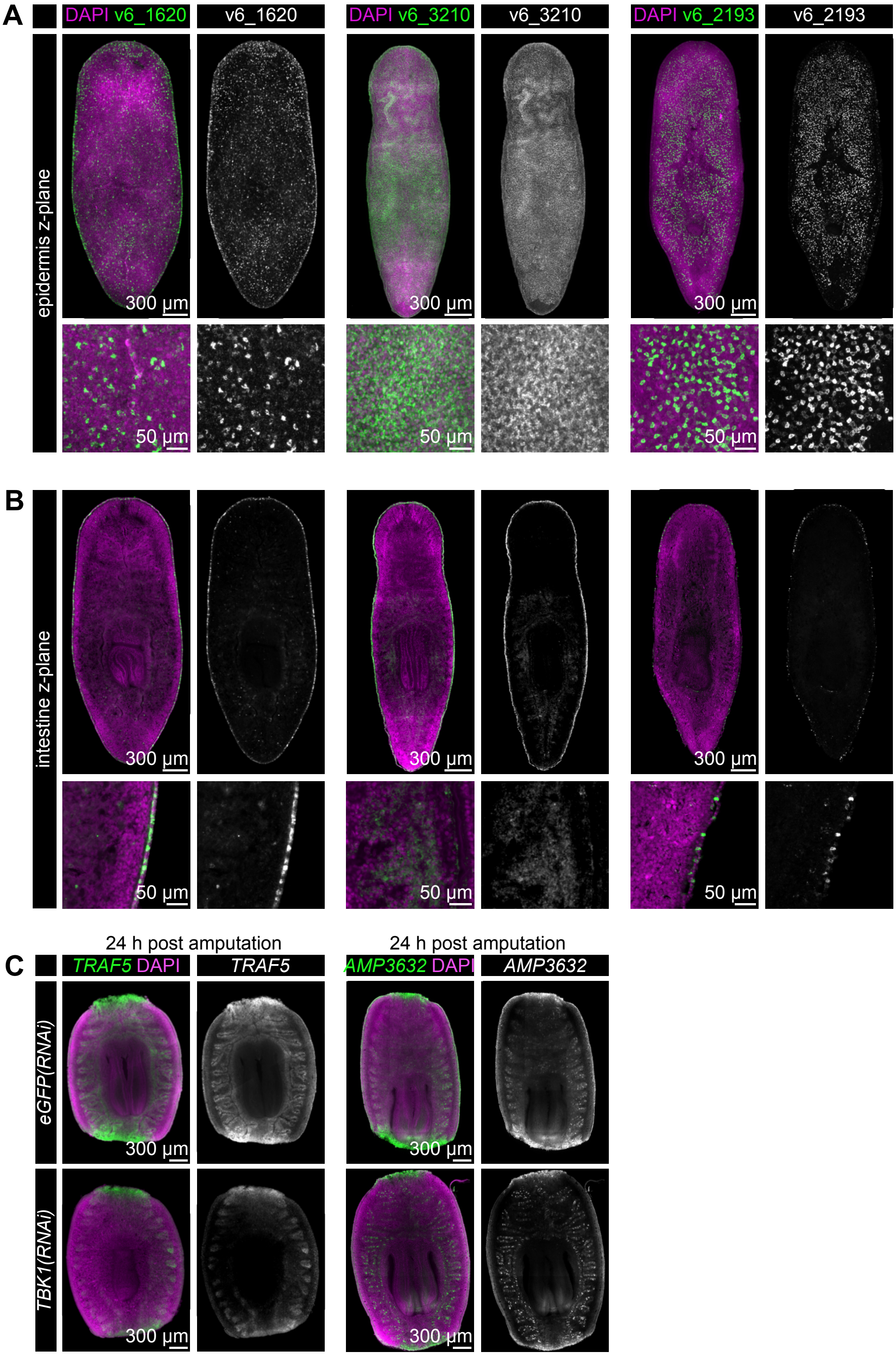

Fig. S5. Expression pattern of transfection response genes

(A-C) Whole mount FISH stainings of indicated gene transcripts in wildtype worms (A-B) and *eGFP(RNAi)* or *TBK1(RNAi)* worms at 24 h post amputation (C). Different focal planes of the same specimens are shown in A (epidermis focal plane) and B (intestine focal plane).

Development • Supplementary information

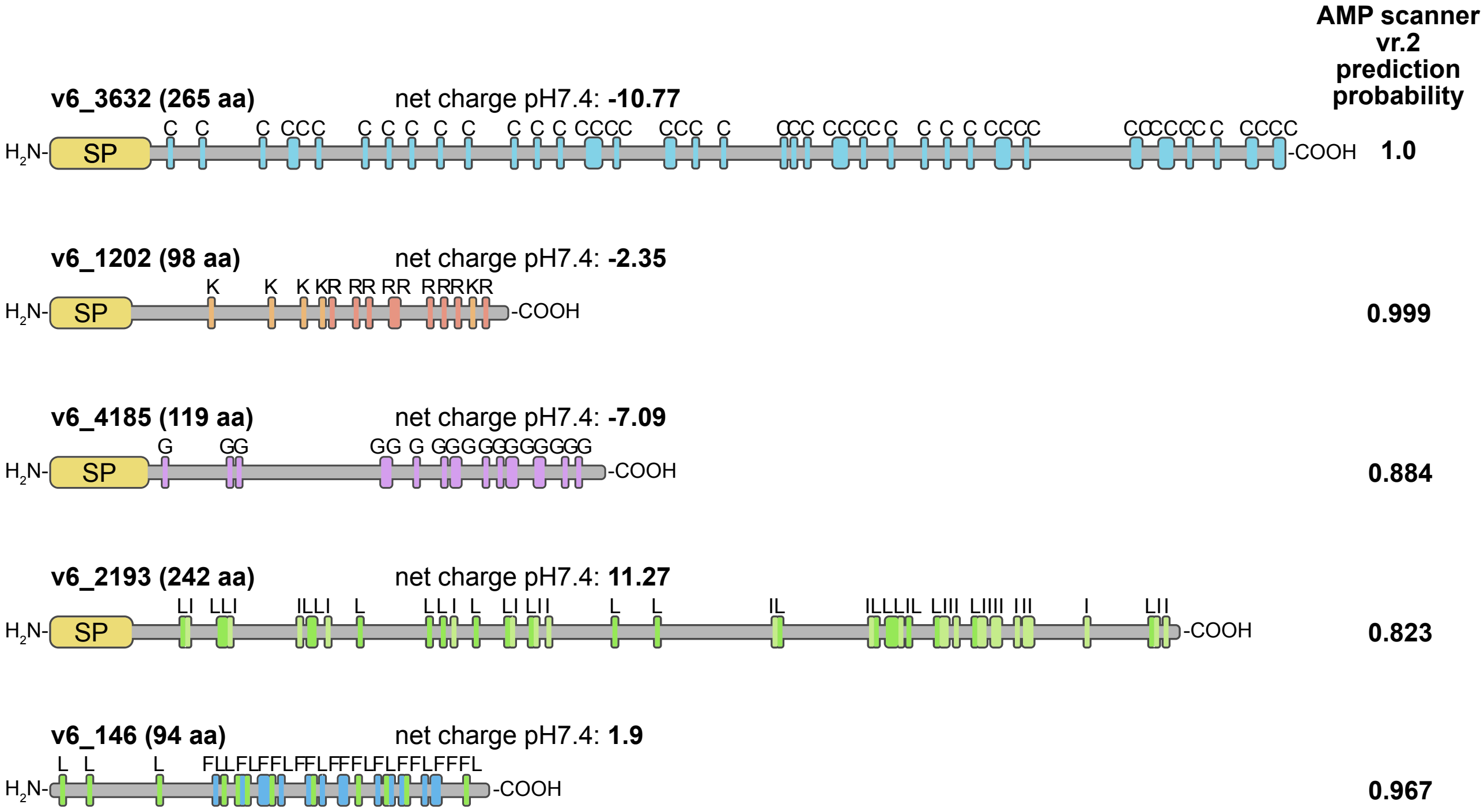

Fig. S6. Properties of predicted antimicrobial peptides

Schematic presentation of predicted antimicrobial peptide (AMP) proteins. Repetitive amino acid sequences, signal peptide predictions (SP) and AMP prediction probability using AMP scanner vr. 2 (Veltri et al. 2018) are indicated.

Development • Supplementary information

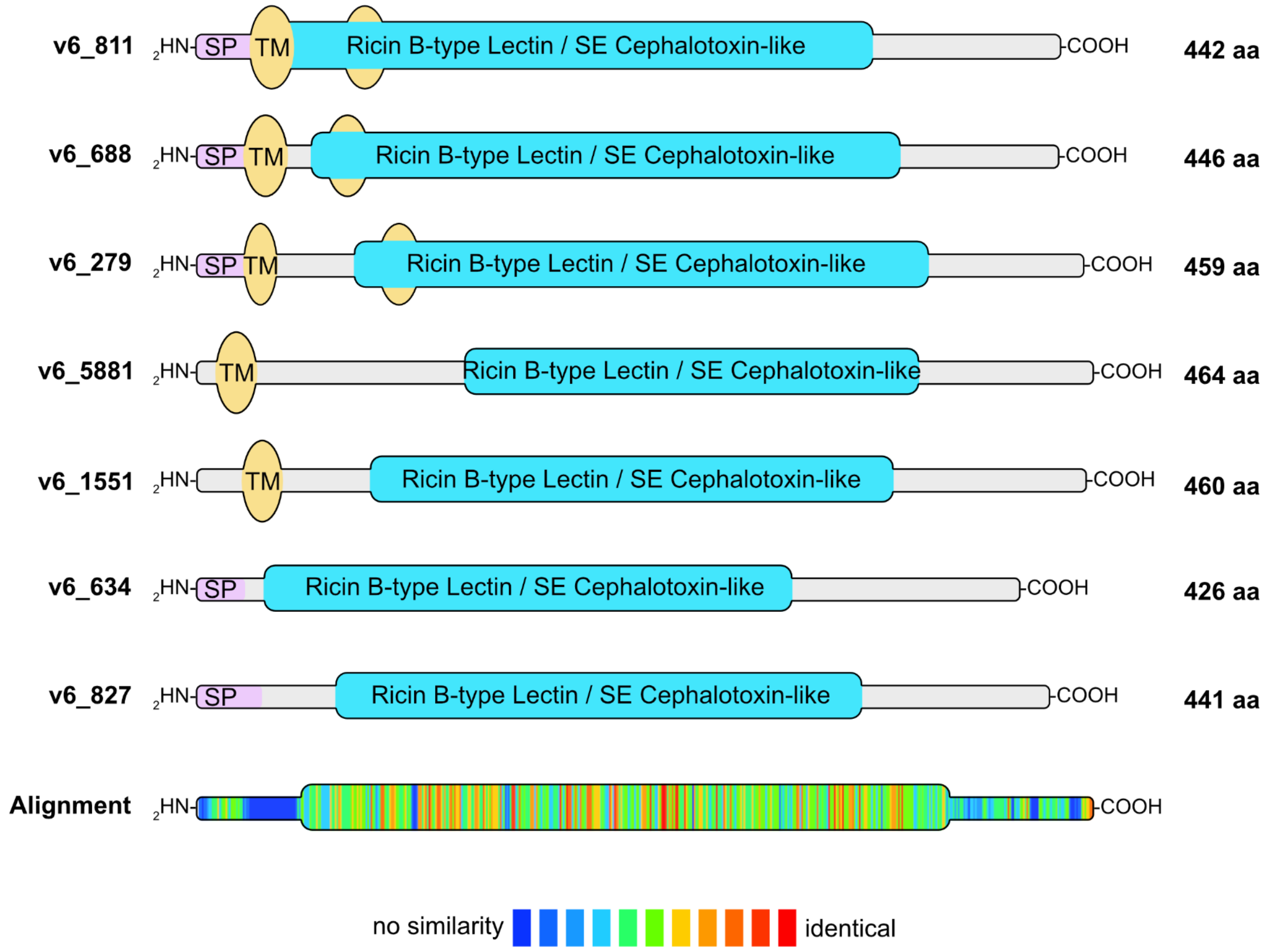

Fig. S7. *Smed* Rapunzel proteins

Schematic presentation of domain structure of Rapunzel (RPZ) proteins. Transcript ID number from dd_Smed_v6 transcriptome, signal peptide prediction (SP), transmembrane domain prediction (TM) and amino acid (aa) number are indicated. A color coded alignment of all RPZ proteins using PRALINE (Simossis and Heringa 2005) shows medium sequence similarity between the proteins.

Development • Supplementary information

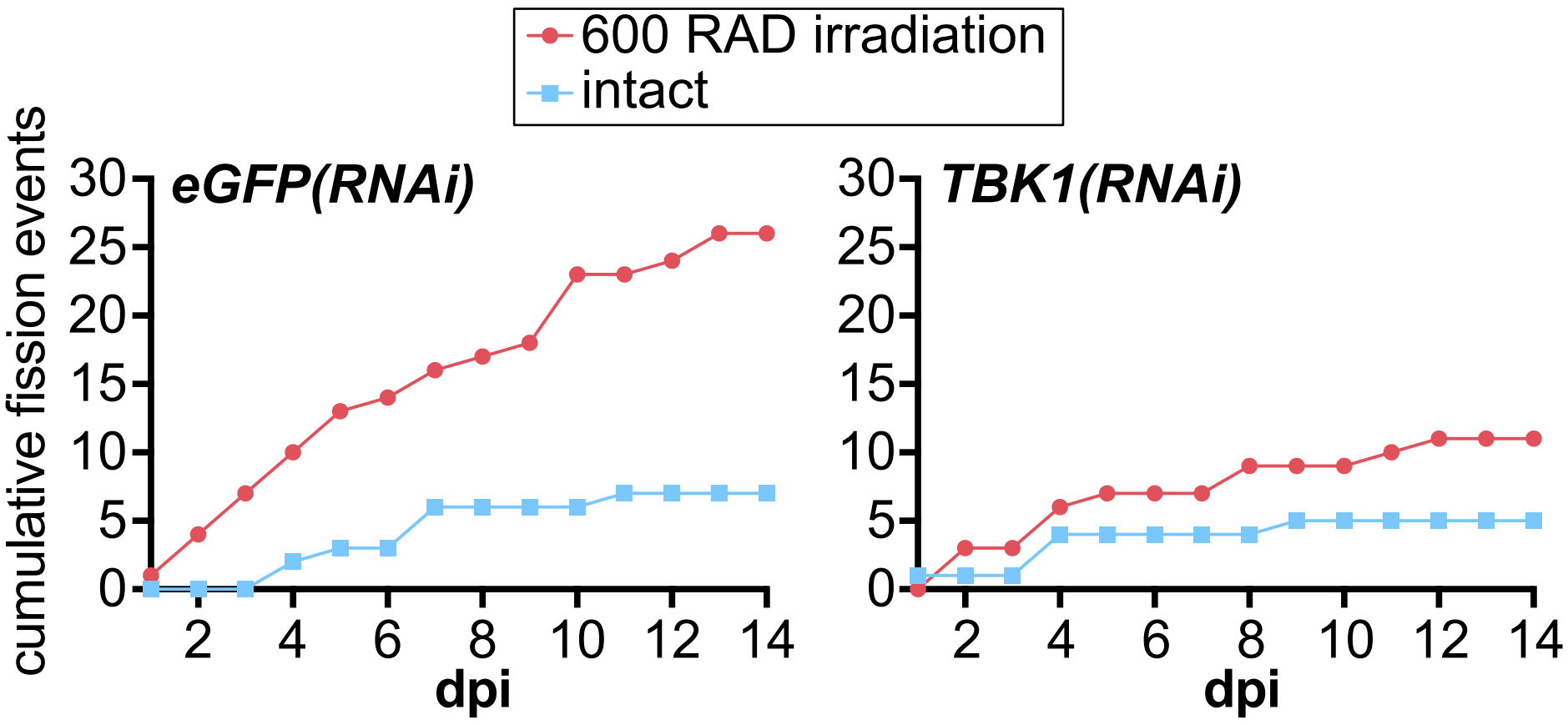

Fig. S8. Fissioning response after irradiation is suppressed in *TBK1(RNAi)* worms

Cumulative events of fissioning in groups of 20 worms *eGFP(RNAi)* and *TBK1(RNAi)* after lethal irradiation shows that irradiation does not cause an increase in fission during knock-down of *TBK1,* as seen in *eGFP(RNAi)* worms.

Table S1. RNAi targeted genes and primers that were used for dsRNA synthesis template cloning

List of genes that were targeted by RNAi in this study. Transcript IDs, homologs based on sequence similarity and primer sequences that were used for cloning DNA templates for dsRNA synthesis are listed.

Available for download at
https://journals.biologists.com/dev/article-lookup/doi/10.1242/dev.205222#supplementary-data

Table S2. Primers used for cloning of genomic regions

Transcript IDs, gene names, identity of cloned regions and primers that were used for amplification of these regions are listed for genes of which regulatory regions were used to construc NanoLuc expression reporter plasmids.

Available for download at
https://journals.biologists.com/dev/article-lookup/doi/10.1242/dev.205222#supplementary-data

Development • Supplementary information

Table S3. Sequence of NanoLuc codon optimized for Smed

The NanoLuc encoding sequence that was codon optimized for Smed is shown.

Available for download at
https://journals.biologists.com/dev/article-lookup/doi/10.1242/dev.205222#supplementary-data

Table S4. 16s rRNA sequences of identified bacterial species

The 16s rRNA sequences that were concatenated from forward and reverse sanger sequencing of 16s rRNA-region PCR products are shown and inferred bacterial species (based on highest similarity in BLAST search) are listed.

Available for download at
https://journals.biologists.com/dev/article-lookup/doi/10.1242/dev.205222#supplementary-data

Table S5. Differentially expressed genes at 24 hours post injection within and between *eGFP(RNAi)* and *TBK1(RNAi)* worms

Gene IDs, log2 fold change, log2 fold change standard error and padjust values are listed for DEGs from indicated comparisons at 24 hpi within and between *eGFP(RNAi)* and *TBK1(RNAi)* worms. Underlying experimental layout is shown in Fig. 3A.

Available for download at
https://journals.biologists.com/dev/article-lookup/doi/10.1242/dev.205222#supplementary-data

Table S6. Differential protein quantification of Biotin IP from worms transfected with Biotinylated DNA vs DNA

Protein IDs, log2 fold change and padjust values are listed for proteins that were identified and quantified by mass spectrometry in Biotin IP samples. Samples from worms that were transfected with Biotin-labeled DNA were compared to samples from worms that were transfected with DNA (control).

Available for download at
https://journals.biologists.com/dev/article-lookup/doi/10.1242/dev.205222#supplementary-data
