# Supplementary material for: Conserved innate immunity components limit transgene expression in adult planarians
Source: Development. 2026 Mar 27;153(6):dev205222. doi: 10.1242/dev.205222 (PMC13563047; doi:10.1242/dev.205222)
Supplement: Supplementary file 2 [file develop-153-205222_review_history.pdf]

Conserved innate immunity components limit transgene expression in adult planarians

Leonard Drees, Daniel Blösel, Johannes Kowalewski, Uri Weill and Jochen C. Rink
DOI: 10.1242/dev.205222

Editor: Kenneth Poss

Review timeline
Original submission: 8 September 2025
Editorial decision: 19 October 2025
First revision received: 27 January 2026
Accepted: 21 February 2026

Original submission

First decision letter

MS ID#: dev.205222

MS TITLE: Conserved innate immunity components limit transgene expression in adult planarians

AUTHORS: Daniel Blösel, Johannes Kowalewski, Uri Weill, Jochen Christian Rink and Leonard Drees

Dear Dr Drees,

I have now received all the referees reports on the above manuscript, and have reached a decision. The referees' comments are appended below, or you can access them online: please go to.

The overall evaluation is positive and we would like to publish a revised manuscript in Development, provided that the referees' comments can be satisfactorily addressed. Please attend to all of the reviewers' comments in your revised manuscript and detail them in your point-by-point response. If you do not agree with any of their criticisms or suggestions explain clearly why this is so. If it would be helpful, you are welcome to contact us to discuss your revision in greater detail. Please send us a point-by-point response indicating your plans for addressing the referees' comments, and we will look over this and provide further guidance.

Reviewer 1

The work of Leonard et al. represents an important step in establishing transgenic lines in planarians by testing delivery and expression of plasmids. The injection of biotin-labeled DNA to pulldown innate immune response proteins is ingenious. The identification of TBK1 and MRC1 as repressors of transgene expression is exciting. The authors continue to profile genes responding to TBK1 RNAi and injection with transcriptome sequencing. They uncovered many conserved genes reported in immune responses in other species or in planarians already. Overall, the study represents an extensive amount of work supporting TBK1's role in innate immune response in planarians.

It is unfortunate that no additional progress in transgenesis was achieved, and that TBK1's role in innate immune response has been reported. These weakness does not diminish my enthusiasm in this work to be published in Development, should the following comments be addressed, which aim to improve novelty, clarity and inclusiveness of the manuscript.

© 2026. Published by The Company of Biologists under the terms of the Creative Commons Attribution License (https://creativecommons.org/licenses/by/4.0/).

Required major revisions:

Line 208-213: Luminescence signals are not commonly used and accessible to labs around the world, though it is useful to use as a proof-of-principle. It is biologically interesting that the transient luminescence signal leads to studies of innate immune response, and data that follow. The technical utility of the authors' approach can be further demonstrated as a tool to screen for promoters. Along this line of thought, demonstration of delivery and expression of fluorescent proteins will have a broader impact and utility. It will be an important contribution if the authors can show/include data that they have identified promoters that drive high expression of fluorescent proteins besides luminescence.

Line 307-311: It is great to learn that TBK1 and MRC1 RNAi increased transgene expression. How about duration? Was the expression extended in RNAi conditions? Fig. 5A showed extended expression of TBK1 RNAi. How about MRC1 RNAi? The same or different? This may help with the understanding of suppressed transgene expression.

Line 361-381: Figure 3C-D, Are the 4 heatmaps part of one heatmap, but separated into 4 sections for data presentation; or they were calculated as four separate heatmaps, with the same color scale? It will be important to know this as it is possible when all four heatmaps were plotted as one with the same scaling, there may be trends of differential gene expression between mock and TBK1 RNAi. Currently, this information is not clear.

The description of this section of the DEG data is a bit confusing. The statement "Irrespectively, the highly significant reduction in the injection response components in TBK1(RNAi) reveals a clear role of the gene in the coordination of this response (Fig 3D)" is not immediately clear to me.

Required minor revisions:

Line 47: In the description of fascinating phenotypic traits in planarians, and life expectancies, it is reasonable to cite Guo et al. 2025 Annual Review of Genetics, and Dai et al. 2025 Nature Aging. "Other fascinating phenotypic traits of planarians include a wide range of body sizes, reproductive strategies and probably life expectancies (Vila-Farré et al. 2023; Drees and Rink 2023)."

Line 54: In the description of annotated chromosome-scale reference genomes, it is reasonable to include citation of Guo et al. 2022 Nature. "The current tool kit for studying this unique biology includes an annotated chromosome-scale reference genomes assemblies (Ivanković et al. 2024)". In addition, there is gramma issue with this sentence.

Line 103: In the description of the innate immune system of planarians, it is reasonable to include citations of Chai et al. 2025. https://www.biorxiv.org/content/10.1101/2025.03.28.645876v2

line 111: the citation of Arnold et al. 2019 in this part of the sentence is inappropriate. In the description of embryogenesis "hindered by the small size of the zygotes, their embedding within compact yolk cell masses inside a rigid egg shell", the following citations need to be included: Davies et al. eLife 2017; Sánchez Alvarado, Current Opinion in Genetics & Development, 2003; Cardona et al. Development Genes and Evolution 2006

<u>Reviewer 2</u>

SUMMARY OF THE ADVANCE MADE IN THIS PAPER AND ITS POTENTIAL SIGNIFICANCE TO THE FIELD

Drees, et al. investigate the mechanisms limiting transgene expression in the planarian Schmidtea mediterranea. This organism has become a useful experimental subject for studying the mechanisms of whole body regeneration, however, its inaccessibility to transgenic tools limits the types of experimental questions that can be addressed.

© 2026. Published by The Company of Biologists under the terms of the Creative Commons Attribution License (https://creativecommons.org/licenses/by/4.0/).

In this important work the authors show that the planarian's innate immune response is a critical factor that limits transgene expression. They first developed a series of plasmid vectors containing 5' and 3' regions from abundantly expressed genes, replacing the protein coding exons with sequences encoding a NanoLuciferase reporter. They tested several different lipofection reagents to identify the most effective one, then showed that they could detect luciferase activity when its expression was driven by the regulatory regions of several different genes abundantly expressed in neoblasts, but not by regulatory sequences promoting expression in differentiated cells. Luciferase expression was shown to be sensitive to irradiation, which eliminates neoblasts, suggesting that they are able to direct transgene expression in neoblasts. This result is an important first step in generating transgenic planarians.

The authors found that DNA transfection was associated with higher rates of fission, suggesting that the planarians were responding systemically to exogenous DNA, so they identified potential conserved innate immune nucleic acid sensing pathways and their effectors in the planarian genome. They also took a biochemical approach to identify proteins that bound to exogenous biotinylated DNA. From these candidates they then performed a targeted RNAi screen to identify gene knockdowns that increased transgene expression, leading to the identification of TBK1 and MRC1 as factors that limit transgene expression.

To explore the mechanisms downstream of TBK1 activity the authors conducted RNAseq analysis of control and TBK1(RNAi) planarians time courses in response to mock injection or DNA transfection. They identified several TBK1-dependent genes involved in the innate immune response, with either local or global responses to injection and showed that TBK1 mediates the planarian's response to exogenous DNA, RNA, and bacterial pathogens. Thus, the planarian's innate immune system is protecting the animal from exogenous DNA, thereby limiting the effectiveness of transgenesis in this organism.

The work has been carefully conducted and thoughtfully presented in this outstanding manuscript. This is an important contribution to the field of planarian biology and innate immunity in invertebrates. I have no major concerns and from my perspective, the paper could be accepted as is and the sooner it appears in print, the better.

SUGGESTIONS TO AUTHORS

I have a few minor suggestions, which the authors may wish to consider as they revise this.

1. The observation that RPZ4 protein can be detected outside of cells expressing rpz4 RNA in response to DNA transfection and is TBK1 dependent is interesting and potentially important. Because the title of the figure legend makes no mention of this result ("TBK1 regulates the transcription of genes with links to innate immunity"), this interesting result winds up seeming a little buried. Perhaps it's worth its own figure (or at least a more complete legend title)? Does mock injection lead to a similar result or is this also dependent on exogenous DNA? This is the one scientific question that should probably be answered to make sure that the model in Fig. 6 is correct, is Rapunzel release due to a nucleic acid sensor, or is it a response to wounding?

2. There is some concern that depending on the final size of the figures in the journal, some panels may be difficult for readers to read or clearly distinguish. For example, the inset in Fig. 2E might benefit from a horizontal orientation that would allow the text and images to be increased in size. Similarly, it is difficult to distinguish the different experimental parameters in Figs. 3E, 5C, and 5E, because they are marked with black lines and very small distinguishing shapes. Perhaps it would be preferable to also use different colors to help the reader better see the data (as in Fig. 5A)?

3. It might be helpful to the reader if all the genes shown in Fig. 3E were also identified in the heat maps depicted in Fig. 3C. Several predicted AMPs are unlabeled, so the correspondence with 3E is unclear.

4. Line 489: serendipity -> serendipitously

© 2026. Published by The Company of Biologists under the terms of the Creative Commons Attribution License (https://creativecommons.org/licenses/by/4.0/).

Reviewer 3

SUMMARY OF THE ADVANCE MADE IN THIS PAPER AND ITS POTENTIAL SIGNIFICANCE TO THE FIELD

Drees and colleagues tackle the vexing problem of optimizing transgenesis in the planarian S. mediterranea and begin to uncover the biological basis for suppression of transgene expression. Importantly, they provide the first demonstration of transient transfection of a NanoLuc reporter plasmid, flanked by native 5'UTR and 3'UTR sequences from ubiquitously expressed or neoblast-enriched S. mediterranea genes, providing a needed positive control and baseline for future optimization experiments. Unfortunately, this technique did not yield detectable fluorescent reporter expression, demonstrating the need for continued investment in development of this technique. Impressively, the authors began investigating the innate immune-based mechanisms that sense foreign nucleic acids and mediate transgene suppression. Using biotin-labeled DNA, the authors performed streptavidin-mediated pulldowns of foreign DNA binding partners, followed by mass spectrometry, as well as identification of candidate innate immunity regulators, leading to the identification of Tank Binding Kinase-1 (TBK-1), a regulator of local and systemic responses to plasmid DNA injection. The authors provide convincing evidence of TBK-1-dependent suppression of transgene expression, as well as dependence of local and systemic aspects of the response, including expression of stress/wound-responsive genes locally at the transfection site (24 hpi), and gut-wide expression of wound-responsive genes. Intriguingly, DNA injection triggers fission in a TBK-1-dependent manner. Much remains to be determined about upstream sensing of DNA, and downstream mediators of the TBK-1-dependent response; this will require further study and some ingenuity given that some of the deeply conserved nucleic acid sensors and transcriptional effectors of Toll/NF-KB signaling are not present, or are highly divergent and without functional roles, in S. mediterranea.

This high-quality work was performed rigorously and is presented clearly. The work is appropriate for publication in Development; it will be necessary and informative for planarian biologists as well as others working to optimize transgenesis in emerging research organisms. There are several intriguing leads here and certainly, more to do in the future, but the manuscript stands as a publishable unit. My comments are minor and will not require new experiments. I commend the authors for undertaking a notoriously difficult subject of study for the benefit of the community. We need to continue and to reward incremental progress and to understand more about the biological basis of negative results. I enjoyed this study and the novel findings about the innate immune response, and congratulate you on a job well done here.

SUGGESTIONS TO AUTHORS

Comments (experiments not necessary for publication, but could be mentioned further in the discussion):
Have the authors attempted transfection of a fluorescently labeled plasmid to look at its localization within transfected cells, and perdurance over time? This would help to address whether cell division is necessary for nuclear entry, for example, and may help to explain why signal was not detected for the muscle- and epidermal-driven constructs.

Have the authors attempted transfection of plasmid DNA into sexually reproducing or pseudo-sexual planarians (S. mediterranea, or other species)? I would be curious to know whether germ cells (either sex, any stage) are amenable to transient transfection (using the ubiquitously expressed promoter-driven transgenes).

Have the authors used Trans-IT-X2 or related reagents for delivery of Cas9 and sgRNAs to determine whether gene editing can occur in neoblasts?

Methods section:
It's very interesting that so many conserved pathways that sense foreign nucleic acids are seemingly absent from S. mediterranea. You mention that the description of your search appears in the Methods section (likely in the software, algorithms, and repositories), but the details are sparse. Do you have a supplementary table that would summarize the queries run? Does Figure 2A encompass the summarized data?

© 2026. Published by The Company of Biologists under the terms of the Creative Commons Attribution License (https://creativecommons.org/licenses/by/4.0/).

Mass spectrometry data analysis: what asexual Smed proteome was used?

Figure 3B/Methods - The 3B legend is also incomplete (it doesn't mention the eGFP(RNAi) 24 hpi vs TBK1(RNAi) 24 hpi data). The main text should also call out Supplementary Table 5. Providing annotation (homologs, functional domains, or the like) for the injection- and TBK-1-dependent DEGs would be a great aid to readers perusing these files.

First revision

Author response to reviewers' comments

Reviewer 1:

The work of Leonard et al. represents an important step in establishing transgenic lines in planarians by testing delivery and expression of plasmids. The injection of biotin-labeled DNA to pulldown innate immune response proteins is ingenious. The identification of TBK1 and MRC1 as repressors of transgene expression is exciting. The authors continue to profile genes responding to TBK1 RNAi and injection with transcriptome sequencing. They uncovered many conserved genes reported in immune responses in other species or in planarians already. Overall, the study represents an extensive amount of work supporting TBK1's role in innate immune response in planarians.

It is unfortunate that no additional progress in transgenesis was achieved, and that TBK1's role in innate immune response has been reported.

These weakness does not diminish my enthusiasm in this work to be published in Development, should the following comments be addressed, which aim to improve novelty, clarity and inclusiveness of the manuscript.

We thank Reviewer 1 for the optimistic feedback. While we agree that the role of TBK1 in the immune response of vertebrate and some invertebrates has been reported, we want to emphasize that we provide here first evidence that TBK1 has a role in the innate immune response of planaria, an animal in which mechanisms of innate immunity are mostly unknown.

Required major revisions:

(1)
Line 208-213: Luminescence signals are not commonly used and accessible to labs around the world, though it is useful to use as a proof-of-principle. It is biologically interesting that the transient luminescence signal leads to studies of innate immune response, and data that follow. The technical utility of the authors' approach can be further demonstrated as a tool to screen for promoters. Along this line of thought, demonstration of delivery and expression of fluorescent proteins will have a broader impact and utility. It will be an important contribution if the authors can show/include data that they have identified promoters that drive high expression of fluorescent proteins besides luminescence.

We fully agree with the reviewer, that high-level expression of fluorescent proteins in Smed would be very useful. However, as we clearly state in the text, we are so far not able to detect eGFP, mCherry or mScarlet fluorescence above background with any of the promoter constructs tested for NanoLuc expression. These experiments are described in lines 191-196 and in lines 268-271 and 555-558, including the explicit statement that we were not able to achieve detectable expression levels of fluorescent proteins.
Nevertheless, we are of the opinion that our unambiguous detection of plasmid-based reporter expression and the identification of endogenous innate immunity barriers to transgene expression represent important progress towards the development of high-level transgene expression and we hope that our manuscript will inspire others in the planarian research community to push towards this goal.

© 2026. Published by The Company of Biologists under the terms of the Creative Commons Attribution License (https://creativecommons.org/licenses/by/4.0/).

(2)
Line 307-311: It is great to learn that TBK1 and MRC1 RNAi increased transgene expression. How about duration? Was the expression extended in RNAi conditions? Fig. 5A showed extended expression of TBK1 RNAi. How about MRC1 RNAi? The same or different? This may help with the understanding of suppressed transgene expression.

Thank you. We have added the requested expression time course under *MRC1(RNAi)* and added the data in Fig. 5A. Accordingly, we changed the text in lines 396-408 to: “To delineate the time window during which TBK1 and MRC1 affect transgene expression, we performed time-course experiments comparing NanoLuc reporter signal dynamics after plasmid injection into control (*eGFP(RNAi)*), *TBK1(RNAi)*, or *MRC1(RNAi)* worms. In *TBK1(RNAi)* worms, both mean expression levels above background and the number of expressing worms were increased at all tested time points (Fig. 5A); moreover, the signal remained detectable at 10 dpi, unlike in controls (Fig. 5A). In contrast, *MRC1(RNAi)* elevated the NanoLuc signal only at 4 and 6 dpi, with no worms exceeding *eGFP(RNAi)* levels at later time points (Fig. 5A). Elucidating whether TBK1 and MRC1 affect transfection efficiency, reporter stability, or survival of expressing cells will require the development of reporter constructs enabling direct visualization of transfected cells (see Discussion). Given TBK1’s stronger effect on NanoLuc expression, we focused subsequent experiments on its roles in immunity and stress responses.”

(3)
Line 361-381: Figure 3C-D, Are the 4 heatmaps part of one heatmap, but separated into 4 sections for data presentation; or they were calculated as four separate heatmaps, with the same color scale? It will be important to know this as it is possible when all four heatmaps were plotted as one with the same scaling, there may be trends of differential gene expression between mock and TBK1 RNAi. Currently, this information is not clear.

The heatmaps in Figure 3C-D were calculated with the same color scale so that color intensities are comparable across all panels. We agree with the reviewer that this information is not clearly stated in the text. Therefore, we changed lines 308-313 to:
“Plotting the expression levels of injection-response genes (DEGs at 24 hpi) from the *eGFP(RNAi)* group across time points after injection showed that their expression returned to baseline by 72 hpi (Fig. 3C). These genes also behaved similarly in *TBK1(RNAi)* worms, also returning to baseline by 72 hpi (Fig. 3C–D); however, in contrast to *eGFP(RNAi)*, many of these genes did not significantly diverge from baseline at 24 hpi in *TBK1(RNAi)* worms (Fig. 3C–D).”

(4)
The description of this section of the DEG data is a bit confusing. The statement "Irrespectively, the highly significant reduction in the injection response components in TBK1(RNAi) reveals a clear role of the gene in the coordination of this response (Fig 3D)" is not immediately clear to me.

We agree that the description of the effect of TBK1 on changes in gene expression is unclear in this section. We changed the text in lines 321-323 to: “Irrespectively, the significantly reduced changes in gene expression after injection in *TBK1(RNAi)* worms compared to *eGFP(RNAi)* worms reveal a clear role of TBK1 in coordination of the transcriptional response to injection (Fig 3D).”

Required minor revisions:

(5)
Line 47: In the description of fascinating phenotypic traits in planarians, and life expectancies, it is reasonable to cite Guo et al. 2025 Annual Review of Genetics, and Dai et al. 2025 Nature Aging. "Other fascinating phenotypic traits of planarians include a wide range of body sizes, reproductive strategies and probably life expectancies (Vila-Farré et al. 2023; Drees and Rink 2023)."

We included the Guo et al. review reference, but don’t consider the additional citation of Dai et al. as required.

(6)

© 2026. Published by The Company of Biologists under the terms of the Creative Commons Attribution License (https://creativecommons.org/licenses/by/4.0/).

Line 54: In the description of annotated chromosome-scale reference genomes, it is reasonable to include citation of Guo et al. 2022 Nature. "The current tool kit for studying this unique biology includes an annotated chromosome-scale reference genomes assemblies (Ivanković et al. 2024)". In addition, there is gramma issue with this sentence.

We corrected the grammar of the sentence. However, the sentence refers to the “annotated choromosome scale reference genome assembly”. The ENSEMBL *S. mediterranea* reference genome annotations are described in the cited reference. Hence we don’t see the need for citing either earlier versions of the genome assembly or earlier annotation versions.

(7)
Line 103: In the description of the innate immune system of planarians, it is reasonable to include citations of Chai et al. 2025. https://www.biorxiv.org/content/10.1101/2025.03.28.645876v2

We thank the reviewer for the suggestion and added the citation, together with the text: “…a specialized cell type called “Ruptoblasts” was identified to be involved in immune defense (Chai et al. 2025)…”

(8)
line 111: the citation of Arnold et al. 2019 in this part of the sentence is inappropriate. In the description of embryogenesis "hindered by the small size of the zygotes, their embedding within compact yolk cell masses inside a rigid egg shell", the following citations need to be included: Davies et al. eLife 2017; Sánchez Alvarado, Current Opinion in Genetics & Development, 2003; Cardona et al. Development Genes and Evolution 2006

We thank the reviewer for the suggestion and swapped out Arnold et al. 2019 with the suggested references.

Reviewer 2:

SUMMARY OF THE ADVANCE MADE IN THIS PAPER AND ITS POTENTIAL SIGNIFICANCE TO THE FIELD

Drees, et al. investigate the mechanisms limiting transgene expression in the planarian Schmidtea mediterranea. This organism has become a useful experimental subject for studying the mechanisms of whole body regeneration, however, its inaccessibility to transgenic tools limits the types of experimental questions that can be addressed.

In this important work the authors show that the planarian's innate immune response is a critical factor that limits transgene expression. They first developed a series of plasmid vectors containing 5' and 3' regions from abundantly expressed genes, replacing the protein coding exons with sequences encoding a NanoLuciferase reporter. They tested several different lipofection reagents to identify the most effective one, then showed that they could detect luciferase activity when its expression was driven by the regulatory regions of several different genes abundantly expressed in neoblasts, but not by regulatory sequences promoting expression in differentiated cells. Luciferase expression was shown to be sensitive to irradiation, which eliminates neoblasts, suggesting that they are able to direct transgene expression in neoblasts. This result is an important first step in generating transgenic planarians.

The authors found that DNA transfection was associated with higher rates of fission, suggesting that the planarians were responding systemically to exogenous DNA, so they identified potential conserved innate immune nucleic acid sensing pathways and their effectors in the planarian genome. They also took a biochemical approach to identify proteins that bound to exogenous biotinylated DNA. From these candidates they then performed a targeted RNAi screen to identify gene knockdowns that increased transgene expression, leading to the identification of TBK1 and MRC1 as factors that limit transgene expression.

To explore the mechanisms downstream of TBK1 activity the authors conducted RNAseq analysis of control and TBK1(RNAi) planarians time courses in response to mock injection or DNA transfection. They identified several TBK1-dependent genes involved in the innate immune response, with either

© 2026. Published by The Company of Biologists under the terms of the Creative Commons Attribution License (https://creativecommons.org/licenses/by/4.0/).

local or global responses to injection and showed that TBK1 mediates the planarian's response to exogenous DNA, RNA, and bacterial pathogens. Thus, the planarian's innate immune system is protecting the animal from exogenous DNA, thereby limiting the effectiveness of transgenesis in this organism.

The work has been carefully conducted and thoughtfully presented in this outstanding manuscript. This is an important contribution to the field of planarian biology and innate immunity in invertebrates. I have no major concerns and from my perspective, the paper could be accepted as is and the sooner it appears in print, the better.

Thank you!

SUGGESTIONS TO AUTHORS

I have a few minor suggestions, which the authors may wish to consider as they revise this.

1. The observation that RPZ4 protein can be detected outside of cells expressing rpz4 RNA in response to DNA transfection and is TBK1 dependent is interesting and potentially important. Because the title of the figure legend makes no mention of this result ("TBK1 regulates the transcription of genes with links to innate immunity"), this interesting result winds up seeming a little buried. Perhaps it's worth its own figure (or at least a more complete legend title)? Does mock injection lead to a similar result or is this also dependent on exogenous DNA? This is the one scientific question that should probably be answered to make sure that the model in Fig. 6 is correct, is Rapunzel release due to a nucleic acid sensor, or is it a response to wounding?

We changed the Figure title to “TBK1 regulates the transcription of genes with links to innate immunity and Rapunzel protein localization”. We furthermore added panels of *rpz4* FISH and anti-RPZ4 immuno stainings from *eGFP(RNAi)* and *TBK1(RNAi)* worms after injection of a mock transfection mix without DNA. We agree with the reviewer that these are important data to add since the we see a similar response in worms that were injected with or without DNA in the transfection mix, suggesting that the TBK1-dependet release of RPZ4 is a wounding response that is not specific to foreign DNA.
We changed the text in lines 384-390 to: “However, 24 h after injection with either a plasmid transfection mix or a mock transfection mix lacking DNA, the RPZ4 antibody additionally labeled small, globular structures near rpz4-expressing cells, although these structures were negative for rpz4 mRNA (Fig. 4B). Interestingly, the appearance of these RPZ4$^+$/rpz4$^-$ structures depended on TBK1, as only the double-positive cells were observed in *TBK1(RNAi)* worms (Fig 4B). These data support a functional link between TBK1 and RPZ4 also in planarians (see below; Lu et al. 2019).”

2. There is some concern that depending on the final size of the figures in the journal, some panels may be difficult for readers to read or clearly distinguish. For example, the inset in Fig. 2E might benefit from a horizontal orientation that would allow the text and images to be increased in size. Similarly, it is difficult to distinguish the different experimental parameters in Figs. 3E, 5C, and 5E, because they are marked with black lines and very small distinguishing shapes. Perhaps it would be preferable to also use different colors to help the reader better see the data (as in Fig. 5A)?

We thank the reviewer for the suggestion to make the figures more accessible and easier to read. We changed the orientation of the inset in Figure 2E to horizontal, color coded experimental conditions in Figure 3E and Figure 5C,E and increased diameter of plot lines and increased the size of text were applicable.

3. It might be helpful to the reader if all the genes shown in Fig. 3E were also identified in the heat maps depicted in Fig. 3C. Several predicted AMPs are unlabeled, so the correspondence with 3E is unclear.

In Fig 3C-D, genes that are differentially expressed at 24 hpi compared to intact worms of the *eGFP(RNAi)* group are shown, while Fig 3E shows genes that are differentially expressed when comparing *TBK1(RNAi)* to *eGFP(RNAi)* at 24 hpi. These sets of genes do not overlap completely and

© 2026. Published by The Company of Biologists under the terms of the Creative Commons Attribution License (https://creativecommons.org/licenses/by/4.0/).

therefore the predicted AMPs that are in both sets are already labeled in both panels. While the expression trend in both sets of genes is very similar, some genes from Fig 3E are just below the significance threshold in the set of genes from Fig 3C-D.

4. Line 489: serendipity -> serendipitously

Changed in text

Reviewer 3:

SUMMARY OF THE ADVANCE MADE IN THIS PAPER AND ITS POTENTIAL SIGNIFICANCE TO THE FIELD

Drees and colleagues tackle the vexing problem of optimizing transgenesis in the planarian S. mediterranea and begin to uncover the biological basis for suppression of transgene expression. Importantly, they provide the first demonstration of transient transfection of a NanoLuc reporter plasmid, flanked by native 5'UTR and 3'UTR sequences from ubiquitously expressed or neoblast-enriched S. mediterranea genes, providing a needed positive control and baseline for future optimization experiments. Unfortunately, this technique did not yield detectable fluorescent reporter expression, demonstrating the need for continued investment in development of this technique. Impressively, the authors began investigating the innate immune-based mechanisms that sense foreign nucleic acids and mediate transgene suppression. Using biotin-labeled DNA, the authors performed streptavidin-mediated pulldowns of foreign DNA binding partners, followed by mass spectrometry, as well as identification of candidate innate immunity regulators, leading to the identification of Tank Binding Kinase-1 (TBK-1), a regulator of local and systemic responses to plasmid DNA injection. The authors provide convincing evidence of TBK-1-dependent suppression of transgene expression, as well as dependence of local and systemic aspects of the response, including expression of stress/wound-responsive genes locally at the transfection site (24 hpi), and gut-wide expression of wound-responsive genes. Intriguingly, DNA injection triggers fission in a TBK-1-dependent manner. Much remains to be determined about upstream sensing of DNA, and downstream mediators of the TBK-1-dependent response; this will require further study and some ingenuity given that some of the deeply conserved nucleic acid sensors and transcriptional effectors of Toll/NF-KB signaling are not present, or are highly divergent and without functional roles, in S. mediterranea.

This high-quality work was performed rigorously and is presented clearly. The work is appropriate for publication in Development; it will be necessary and informative for planarian biologists as well as others working to optimize transgenesis in emerging research organisms. There are several intriguing leads here and certainly, more to do in the future, but the manuscript stands as a publishable unit. My comments are minor and will not require new experiments. I commend the authors for undertaking a notoriously difficult subject of study for the benefit of the community. We need to continue and to reward incremental progress and to understand more about the biological basis of negative results. I enjoyed this study and the novel findings about the innate immune response, and congratulate you on a job well done here.

Thank you!

SUGGESTIONS TO AUTHORS

Comments (experiments not necessary for publication, but could be mentioned further in the discussion):

(1)
Have the authors attempted transfection of a fluorescently labeled plasmid to look at its localization within transfected cells, and perdurance over time? This would help to address whether cell division is necessary for nuclear entry, for example, and may help to explain why signal was not detected for the muscle- and epidermal-driven constructs.

© 2026. Published by The Company of Biologists under the terms of the Creative Commons Attribution License (https://creativecommons.org/licenses/by/4.0/).

Thank you for the suggestion. We have now added experiments in which we used Digoxigenin(DIG)-labeled plasmid for injections with and without transfection reagent, followed by fixation of injected worms at different time points after injection and immunostaining using anti-DIG antibody. Interestingly, the most prominent effect of the TransIT-X2 transfection reagent was to limit the dispersion of the plasmid DNA after injection. Furthermore, we found that most of the DIG-labeled plasmid DNA is degraded within 24 hours after injection. However, whether the detected DIG signal is within or outside of cells is inherently difficult to ascertain. We therefore integrated these data into the supplementary material (Fig S1). Following text was added in lines 157-161: “Initial transfections of Digoxigenin-labeled plasmid with and without transfection reagent revealed that transfection reagent restricts dispersion and keeps the plasmid concentrated at the injection site (Fig S1A). However, the rapid loss of the DIG-DNA signal within 24 h, irrespective of the presence of transfection reagent, suggests the rapid degradation of the bulk of injected plasmid (Fig S1B).”

(2)
Have the authors attempted transfection of plasmid DNA into sexually reproducing or pseudo-sexual planarians (S. mediterranea, or other species)? I would be curious to know whether germ cells (either sex, any stage) are amenable to transient transfection (using the ubiquitously expressed promoter-driven transgenes).

Thank you for this suggestion. We have not carried out these experiments so far, because our sexual *S. mediterranea* are currently practically infertile and for the fertile species in our collection, we do not yet have genome assemblies for reporter construct assembly. But this is an interesting experiment to try in the future.

(3)
Have the authors used Trans-IT-X2 or related reagents for delivery of Cas9 and sgRNAs to determine whether gene editing can occur in neoblasts?

Again, thank you for this suggestion. However, such experiments need robust methods to detect and maintain gene editing events which is complicated by the fact that gene editing in few neoblasts would create a chimeric animal with the majority of neoblasts being wildtype. Such methods need to be established in order to quantify gene editing events and optimize methods which was not in the scope of this publication.

Methods section:

(4)
It's very interesting that so many conserved pathways that sense foreign nucleic acids are seemingly absent from S. mediterranea. You mention that the description of your search appears in the Methods section (likely in the software, algorithms, and repositories), but the details are sparse. Do you have a supplementary table that would summarize the queries run? Does Figure 2A encompass the summarized data?

Figure 2A encompasses the summarized data in the sense that clearly conserved components showed highest similarities in BLAST queries from *Drosophila* to Smed and human to Smed while components labeled as absent gave either no result or conflicting results in BLAST queries. We added following text in lines 755-757: “Proteins were labeled as “conserved in Smed” when queries from *Drosophila* and human proteins showed highest similarity to the same planarian protein, and “absent in Smed” when no Smed protein with sequence similarities was detectable.”

(5)
Mass spectrometry data analysis: what asexual Smed proteome was used?

We added further description of the used proteome in lines 731-733: “Raw data were analysed with MaxQuant (version 1.6.17.0) using a merged *Smed* proteome from longest ORFs for each gene of

© 2026. Published by The Company of Biologists under the terms of the Creative Commons Attribution License (https://creativecommons.org/licenses/by/4.0/).

dd_Smed_v6 and dd_Smes_v2 as a sequence database. Sequences with more than 95 % identity were merged."

(6)
Figure 3B/Methods - The 3B legend is also incomplete (it doesn't mention the eGFP(RNAi) 24 hpi vs TBK1(RNAi) 24 hpi data). The main text should also call out Supplementary Table 5. Providing annotation (homologs, functional domains, or the like) for the injection- and TBK-1-dependent DEGs would be a great aid to readers perusing these files.

Thank you for the suggestions. We completed the legend of Figure 3B, added a reference to Table S5 in lines 302 and 308 and added a sheet tab to table S5 with annotations of the DEGs between *TBK1(RNAi)* and *eGFP(RNAi)* at 24 hpi.

Second decision letter

MS ID#: dev.205222R1

MS TITLE: Conserved innate immunity components limit transgene expression in adult planarians

AUTHORS: Daniel Blösel, Johannes Kowalewski, Uri Weill, Jochen Christian Rink and Leonard Drees
ARTICLE TYPE: Research Article

Dear Dr Drees,

I am happy to tell you that your manuscript has been accepted for publication in Development, pending our standard publication integrity checks.

Reviewer 1

Congratulations to the authors! I have no additional comments.

Reviewer 3

SUMMARY OF THE ADVANCE MADE IN THIS PAPER AND ITS POTENTIAL SIGNIFICANCE TO THE FIELD

The authors have satisfactorily acknowledged and addressed all reviewer comments and suggestions. I support publication of the manuscript in Development and congratulate the authors on their contributions!

© 2026. Published by The Company of Biologists under the terms of the Creative Commons Attribution License (https://creativecommons.org/licenses/by/4.0/).
